# Expanding ART for Treatment and Prevention of HIV in South Africa: Estimated Cost and Cost-Effectiveness 2011-2050

**DOI:** 10.1371/journal.pone.0030216

**Published:** 2012-02-13

**Authors:** Reuben Granich, James G. Kahn, Rod Bennett, Charles B. Holmes, Navneet Garg, Celicia Serenata, Miriam Lewis Sabin, Carla Makhlouf-Obermeyer, Christina De Filippo Mack, Phoebe Williams, Louisa Jones, Caoimhe Smyth, Kerry A. Kutch, Lo Ying-Ru, Marco Vitoria, Yves Souteyrand, Siobhan Crowley, Eline L. Korenromp, Brian G. Williams

**Affiliations:** 1 HIV/AIDS Department, World Health Organization, Geneva, Switzerland; 2 Philip R. Lee Institute for Health Policy Studies, University of California San Francisco, San Francisco, California, United States of America; 3 Huntingdon, England, United Kingdom; 4 Office of the United States Global AIDS Coordinator, Department of State, Washington, D.C., United States of America; 5 Vestergaard Frandsen, Lausanne, Switzerland; 6 South African National AIDS Council, Johannesberg, South Africa; 7 Department of Epidemiology, University of North Carolina, Chapel Hill, North Carolina, United States of America; 8 Global Fund to Fight AIDS, Tuberculosis, and Malaria, Geneva, Switzerland; 9 Department of Public Health, Erasmus MC, University Medical Center Rotterdam, Rotterdam, The Netherlands; 10 South African Centre for Epidemiological Modelling and Analysis, Stellenbosch, South Africa; Jiangsu University, China

## Abstract

**Background:**

Antiretroviral Treatment (ART) significantly reduces HIV transmission. We conducted a cost-effectiveness analysis of the impact of expanded ART in South Africa.

**Methods:**

We model a best case scenario of 90% annual HIV testing coverage in adults 15–49 years old and four ART eligibility scenarios: CD4 count <200 cells/mm^3^ (*current practice*), CD4 count <350, CD4 count <500, all CD4 levels. 2011–2050 outcomes include deaths, disability adjusted life years (DALYs), HIV infections, cost, and cost per DALY averted. Service and ART costs reflect South African data and international generic prices. ART reduces transmission by 92%. We conducted sensitivity analyses.

**Results:**

Expanding ART to CD4 count <350 cells/mm^3^ prevents an estimated 265,000 (17%) and 1.3 million (15%) new HIV infections over 5 and 40 years, respectively. Cumulative deaths decline 15%, from 12.5 to 10.6 million; DALYs by 14% from 109 to 93 million over 40 years. Costs drop $504 million over 5 years and $3.9 billion over 40 years with breakeven by 2013. Compared with the *current* scenario, expanding to *<500* prevents an additional 585,000 and 3 million new HIV infections over 5 and 40 years, respectively. Expanding to *all CD4 levels* decreases HIV infections by 3.3 million (45%) and costs by $10 billion over 40 years, with breakeven by 2023. By 2050, using higher ART and monitoring costs, *all CD4 levels* saves $0.6 billion versus *current*; other ART scenarios cost $9–194 per DALY averted. If ART reduces transmission by 99%, savings from *all CD4 levels* reach $17.5 billion. Sensitivity analyses suggest that poor retention and predominant acute phase transmission reduce DALYs averted by 26% and savings by 7%.

**Conclusion:**

Increasing the provision of ART to <350 cells/mm3 may significantly reduce costs while reducing the HIV burden. Feasibility including HIV testing and ART uptake, retention, and adherence should be evaluated.

## Introduction

After over 30 years, we still find ourselves struggling to address a Human Immunodeficiency Virus (HIV) pandemic in which over 30 million people have died [1] [2]. In 2010, estimated 34 million people were living with HIV with 67% in sub-Saharan Africa [3]. Although approximately 47% of people who need treatment are getting it, universal access to treatment still remains a dream for millions [3], [4]. By the end of 2010, 6.6 million people were on antiretroviral treatment (ART), but 7.6 million in need lacked access, and in 2010 there were an estimated 2.7 million new HIV infections [3] [4] [5]. Without substantial improvements in prevention, we are unlikely to meet access targets for life-saving ART with a projected 47.5 million cumulative HIV infections by 2031 [6]. Yet stopping the HIV epidemic has proven elusive in most settings. Behavioral strategies have failed to control generalized epidemics, despite evidence of good effectiveness for strategies targeted to high risk situations [7], [8]. Biomedical interventions have been disappointing; one review found that out of 27 randomized controlled trials including vaccines, microbicides, and herpes suppression trials, 22 failed to show efficacy [9], [10], [11], [12]. Positive trials include three for male circumcision, which in modeling studies shows prevention potential that is large but insufficient to interrupt the epidemic [13]. More recently randomized controlled trials on the use of pre-exposure prophylaxis (PrEP) and ART to prevent HIV transmission in serodiscordant couples have provided positive results [14] [15], [16], [17] and the overall situation has increased interest in the potential prevention role of ART [18], [19].

There is growing scientific evidence that supports the use of ART for the prevention of HIV transmission. Viral load is the greatest risk factor for HIV transmission and lowering the viral load is essential to interrupting transmission [20], [21]. Sexual transmission of HIV-1 is rare among persons with levels of less than 1500 copies of HIV-1 RNA per milliliter [20], [22], [23]. ART dramatically lowers viral load and numerous observational studies have demonstrated its potential for prevention of HIV transmission [22], [24], [25], [26]. In Uganda testing and counseling combined with ART reduced transmission risk by 98% [24]. A 2009 meta-analysis including 11 cohorts (5021 heterosexual couples) found zero risk of sexual transmission while on ART for HIV-1 ribonucleic acid below 400 copies and an overall 92% reduction in transmission risk per person-year for those on ART versus untreated individuals [27]. A 2010 randomized controlled study of genital herpes simplex virus (HSV-1) treatment among HIV-serodiscordant heterosexual couples in Africa found a 92% reduction in transmission if the HIV-positive partner was on ART [22]. In May 2011, the HPTN 052 trial comparing immediate antiretroviral treatment below 550 CD4 mm^3^ with delayed treatment for the HIV positive partners in discordant couples was stopped 4 years early due to compelling evidence that early treatment reduces HIV transmission in discordant couples by 96% [14]. The scientific evidence also suggests a significant community-level impact of ART on HIV transmission and in British Columbia lower HIV incidence among injecting drug users is associated with ART use and a decrease in community plasma HIV-1 RNA concentrations [28]. A 2004 study from Taiwan found a 53% reduction in new HIV cases associated with free access to ART [29]. A recent San Francisco study found that the number of new HIV diagnoses fell by 45% between 2004 and 2008, as average HIV viral load fell by 40% [30].

Models suggest large potential epidemic effects with expanded ART. Early theoretical analyses demonstrated that rapid scale-up of conventional ART approaches could significantly reduce mortality [31] and have a substantial impact on HIV incidence [19], [32]. Others concluded that expanding access to ART could foster the proliferation of ARV-resistant strains [33]. A recent model of the potential impact of ART, using available data and focusing on a generalized heterosexual HIV epidemic in South Africa, found that expanding access to ART for everyone at CD4 count <350 cells/mm^3^ could have a significant impact on morbidity, mortality and HIV incidence. Expanding beyond to all CD4 levels with combined prevention interventions resulted in a 95% reduction in HIV incidence in 10 years [18]. More recently the HIV *Investment Framework Study Group* incorporated the prevention impact of increased access to ART in their investment approach for an effective response to HIV/AIDS [34].

There has been an unprecedented investment in confronting the HIV pandemic–UNAIDS estimated the investment at $13.8 billion in 2008, including half domestic, one third bilateral, one eighth multilateral, and 5% philanthropic and more recently in 2011 at 16.6 billion [35] [34]. More recently, UNAIDS estimates that 22 billion annually will be needed to meet targets including placing 15 million people on ART by 2015 [34] [36]. In 2009 at the L'Aquila Summit, the G8 confirmed its support for achieving universal access to HIV prevention, care and treatment [37]. The global health leadership recently recognized the benefits of earlier ART. In November 2009 the World Health Organization revised its ART guidelines to include a CD4 threshold of 350 cells/mm^3^ [38]. This translated into an estimated 50% increase in the number of people needing ART, compared to need under the pre-2009 guideline of 200 CD4 cells/mm3. Given the current shortfall in ART access, achieving universal access would require a major effort to reach the millions of people who are immunocompromised but not on treatment. The new guidelines have raised questions regarding the short-term economic feasibility of delivering life-saving ART, and the long-term implications for epidemic trends and costs.

In 2008, South Africa adopted a policy of providing 80% of HIV-infected individuals with care and support [39] and more recently has adjusted its ART guidelines to increase earlier access to treatment [40]. Although some studies have examined financing the HIV/AIDS response, the net economics of expanding ART, have not been formally assessed for Southern Africa. [41] [42] [43] [34], [44] [45] [34], except for one recent study comparing CD4 thresholds <350 cells/mm^3^ with <200 cells/mm^3^ [46]. In the past, the cost-effectiveness of HIV prevention and of treatment have been contrasted, to inform the mix of investment in these apparently distinct activities [47]. This generated considerable controversy, because of the implication that treatment for sick individuals could be displaced in favor of more cost-effective prevention [48]. Expanding access to ART to those earlier in disease in South Africa offers an analytic and policy opportunity to align the dual missions of helping sick individuals and lowering the future societal burden of disease. It also provides insights into the potential health and economic gains from a ‘front loaded’ HIV control investment strategy. The analysis presented here assesses the potential net health system cost, health benefit, and cost-effectiveness of ART for prevention in South Africa.

## Methods

### Overview

This economic analysis adds a detailed health system and costing framework to a previously published [18] and updated epidemic model (see Information S1 document). We examine four ART coverage scenarios, defined by the CD4 level at which ART is offered, up to universal ART. All scenarios include average annual HIV counseling and testing (HCT). Inputs include demographic factors, current HIV epidemiology and ART use, and the utilization and costs of health services off and on of ART. Outcome measures include new HIV infections, deaths, disability adjusted life years (DALYs), and cost. Scenarios are compared incrementally to assess net cost or savings and, for scenarios with a net cost, cost-effectiveness. The time horizon is 40 years (2011–2050), with interim 5 year and annual results. Analyses are repeated for current and enhanced combination HIV prevention. Sensitivity analyses are performed for all input values. HIV infections and deaths are reported in nominal values, without discounting (costs and DALYs are discounted as note below). The HIV testing and ART interventions are assumed to scale up over five years. Sensitivity analyses are performed for all input values, to assess the impact of uncertainty on results. Methods are summarized below, with additional detail in the Information S1.

### ART scenarios

We assume that HIV-infected individuals age 15–49 become eligible for ART following detection of infection through HIV counseling and testing (HCT) and CD4 testing (not done in “*All CD4 scenario*”). HIV progression is followed after age 49. HCT is assumed in the base case to occur annually, covering 90% of adults, with equal access by gender and HIV risk level. If the CD4 criteria is met, ART is started after one month; otherwise CD4 is re-assessed at the next testing cycle.

We assume a well-functioning program and examine ART coverage scenarios representing a wide range of possible strategies. The *Current practice* scenario involves ART initiation at CD4 count <200 cells/mm^3^ (at the time of the analysis South Africa had not switched to <350 eligibility criteria). In South Africa, the reported number of people on ART increased from 296,400 in 2006 to 935,800 or 67% of estimated need as of mid 2010. The *ART for CD4 count <350 cells*/mm^3^ scenario expands coverage to a CD4 initiation level recently recommended in WHO clinical guidelines [38], [49] and partially adopted in South Africa in 2010 [50]. The *ART for CD4 count <500 cells*/mm^3^ scenario expands coverage to a CD4 level that typically marks the first HIV-related decline in immune markers and increased risk of early HIV-related diseases such as TB [51]. The *All CD4 levels* scenario involves offering ART to everyone immediately after diagnosis with HIV irrespective of CD4 count.

In our model, with randomly distributed annual HIV testing, the likelihood of identifying infected and antibody positive individuals is 7.5% per month (90% annual testing target, divided by 12). Although high acute phase transmission would limit ART's effectiveness in blocking transmission our base case is derived from model calibration to South African surveillance data with consequent R0 (reproductive rate) and a relatively lower acute phase risk (see detailed discussion of model calibration in SI section 6.0). In a sensitivity analysis, we also examine the effect of 30% of transmissions occurring during the acute phase.

Use of ART among those offered is modified by several factors. We assume that 8% of individuals refuse the offer of ART, and that drop-out is subsequently 1.5% per annum. These best practice values are based on monitoring data from the national program in Malawi (see SI in Granich et al available from authors on request) [18]. Although retention data from cohorts suggest a 3% annual attrition due to loss to follow up (with a further 2% due to mortality) [52], we use the best practice figure from the Malawi program which benefits from a referral system among numerous clinical sites, thus retaining most individuals who move. We include mortality (from our clinical progression modeling) and evolution to 2nd line regimens (3% per year among those remaining on ART). Individuals on ART survive longer than those not on ART; thus, over several years the percentage on ART rises as compared with the proportion initially eligible and accepting the offer.

### Program description

The best practice program that we costed has three major components: HIV counseling and testing, ART provision, and human rights and community support. The interventions are assumed to scale up over five years.

Voluntary HIV counseling and testing (HCT) is provided through facility-based testing and community campaigns [53], [54]. Twenty percent of HCT is facility-based client- and provider-initiated testing, while community approaches provide access for the general population (72% of testing) and for marginalized and institutionalized groups (8%). HCT is scaled up over five years (from 2011–2016) to 90% and then maintained at that capacity for annual community and facility-based HIV testing, without differences in coverage by gender or risk.

We costed the provision of ART and related services (ambulatory visits and monitoring tests) through existing Department of Health infrastructure (facilities and staff) and costs. We did not estimate a need for added building space, because our analysis suggests that the overall 25% increase in primary health care visits (with a shift from illness episodes to routine ART monitoring) would be balanced by decreased demand on inpatient services. In the base case, we did not assess the cost of training of personnel for integrated management of ART. We conducted a sensitivity analysis on this cost, without assuming any general savings from improved management (SI, section 5.0). We assumed that all care would be delivered through the public sector (i.e., people would use free public ART rather than draw down limited private coverage).

The human rights and community support has multiple components. We allow for patient counselors at each HIV testing site, monitoring in each district, and legal support in each province, overseen by an independent monitoring organization. We cost free transport and nutritional support for undernourished (BMI<18) patients. The training of counselors and other health care workers includes a human rights and community support module.

We did not cost other types of HIV prevention, either current or enhanced, since our goal is to assess the incremental costs and benefits of expanding ART. For our analysis, all benefits and costs derive from HIV testing and ART. We assessed how the prevention context affects ART cost-effectiveness, but not the cost-effectiveness of prevention, such as behavior change and male circumcision.

### Epidemic model and economic methods

The economic portrayal overlays an epidemic model for South Africa with detailed data on health system resources and costs.

The epidemic model is a deterministic compartmental model fitted to the historical HIV epidemic in South Africa [18]. Key inputs are population size (15 and older), background death and birth rate, HIV force of infection (a factor that determines aggregate HIV incidence in susceptibles based on HIV prevalence), and rate of progression to death through four equal duration HIV stages defined by mean CD4 count. Use of ART reduces the rate of progression by 46%. Averted deaths due to ART derive entirely from this slowed progression; we do not move individuals to higher CD4 levels, and we ignore the modest mortality benefits of ART while at higher CD4 counts. The HIV testing and ART interventions are assumed to scale up over five years. Details are available in the original publication and its supplemental information.18 For this analysis, the model was updated for new data on rising ART use (see above) and lower ART effects on force of infection (via transmission efficiency; see below), and refined to examine varied ART coverage strategies (detail is provided in the SI).

The programmatic and economic overlay is structured around a detailed portrayal of geographically-mapped health facilities, service utilization and costs in South Africa, derived from Department of Health publications. We portray utilization of services by facility level and staff type. This is combined with published research on the utilization of health care services by stage of HIV disease and as a function of therapy status, and unit cost data. Detail on unit costs is provided below, and on overall costing in a technical supplement, on line at [*specific URL to be specified before publication*].

### Input values

Key input values are presented in Table 1 (with citations) and below, with added detail in the SI on line.

**Table 1:** Input values.

Input parameter	Baseline value	Range	Source(s)
**Epidemic**			
HIV prevalence	15.1%		[67]
Mean survival HIV infection to death (years)	11	11–14	[18]
Number on ART (mid 2010)	935,828		[82]
Reduction in HIV disease progression rate with ART	0.46		[18]
Reduction in HIV disease progression rate with CTX	0.28		[56]
Reduction in HIV transmission efficiency with ART	0.92	0.85–0.99	[27]
**Health care**			
ART acceptance rate	92%		[18]
Number of inpatient days per year, not on ART	7.1	4.5–9.7	[56], [61]
Number of inpatient days per year, on ART	1.68	1.6–1.8	[61], [62]
Number of visits to PHC per year, not on ART	5.48		[61], [83]
Number of visits to PHC per year, on ART	9.6		[61], [83]
Morbidity reduction on CTX	0.69	0.84–0.53	
Annual HIV counseling and testing	90%(average)	–	[54]
**Costs (US dollars)**			
HCT (HIV counseling and testing)	11.10		[53], [58], [84]
First line ARV drugs (per year)*	188	116–751	[58], [85]
Second line ARV drugs (per year)*	595	332–1167	[85]
Average hospital day inclusive (level 1/general hospital)	80	70–104	[58]
Average outpatient visit (level 1/general)	28	16–28	[58]
ART monitoring tests new on 1^st^ line therapy	34	34–67	[85]–[86],
ART monitoring tests new on 2^nd^ line therapy	31	31–158	[85]–[86],
ART monitoring tests 2^nd^ year on 1^st^ line	24	24–138	[85]–[86],
ART monitoring tests 2^nd^ year on 2^nd^ line	24	24–264	[85]–[86],

*Using first and second line costs the modeled average cost for ARVs (USD per year): Baseline: 256 (range 207–674).

#### Epidemic

HIV prevalence of 15.1% in the 15–49 age group in South Africa comes from UNAIDS reporting. The number on ART has risen sharply in recent years in South Africa, now reported at over 900,000 [55]. Mean survival of 11 years without ART derives from multiple studies, summarized by us previously. Change in survival due to ART is a 2-fold increase in the base case levels reaching a survival that is 3.5-times as high as without ART. The 46% reduction in disease progression for ART derives from our prior review for ART and added information (see SI). 18 A study in Uganda provides the basis for the 28% reduction for cotrimoxazole [56]. The ART-associated reduction in HIV transmission efficiency (in the model reflected in the force of infection) derives from a recent review [27] and cohort study [57], both of which had point estimates of 92%.

#### Health care

We based ART acceptance rate of 92% and annual drop-out (excluding deaths) on data from the best functioning large ART program we could identify, in Malawi. The rate of change in ART regimens derives from a study in Uganda. The number of inpatient days while not on ART and the lower level on ART come from clinical studies in South Africa and Uganda. These studies found reductions of 84% and 60% associated with ART, respectively; we use 76% at baseline. More days and averted days at lower CD4 levels are reported in the SI. Data on primary health care visits comes from the same studies, and on reductions in sickness episodes (i.e., opportunistic infections) from Uganda.

#### Economic

The unit cost of HIV counseling and testing ($11.10) is derived from testing campaigns in Uganda and Kenya, adjusted to South African wage levels, and data on facility-based testing. Costs are in 2009 US$, local prices are converted at the average exchange rate for 2009 (ZAR 7.407 to 1US$), historic prices are inflated at 4.9% p.a., future costs are discounted at 3.5% p.a.

The cost of first line ARV drugs, based on generic 2009 international prices, is $188, and second line is $595, with an average in the model of $256 for the base case. The most common first line regimen, representing 60% of actual use, is tenofovir, lamivudine, and efavirenz ($201); other regiments are reported in Information S1. Much higher ARV costs are also examined, to reflect higher prices paid currently in South Africa. The cost of a level 1 hospital day is $80, based on South Africa-specific data from WHO CHOICE and consistent with other available data but potentially less than HIV-specific inpatient care (SI, section 3.0). ART monitoring costs in the base case includes (usually twice annually): CD4 ($5), FBC ($7), Creatinine ($7), Chol/TG ($5), and (Glucose ($2); the total is $24–31 per year (prices based on programmatic experience of authors CB Holmes and C Serenata). The base case excludes Hepatitis B Surface antigen (HbSag), Viral Load ($40) and Alanine aminotransferase (ALT, $4), which are examined in a high-cost monitoring option in the sensitivity analyses. Local costs are adjusted to 2009 using South African inflation indices.

### Outcome measures

Outcome measures reported for each ART scenario include process indicators, health events, and standardized outcomes for cost-effectiveness. The process indicators are the number and proportion of HIV-infected individuals on ART over time. Health events include new HIV infections, HIV prevalence, and deaths while HIV-infected, by year. These outcomes are not discounted.

Standardized outcomes for cost-effectiveness include cost and disability adjusted life years (DALYs). Cost represents all resources and associated unit costs for HIV counseling and testing, inpatient and ambulatory care while HIV-infected, medications including ART, and laboratory testing to monitor immune status and toxicities. DALYs are a combined measure of changes in years of life due to premature mortality, and the disability associated with illness. By definition, they represent the burden of disease, and thus are averted by effective interventions. For HIV prevention and life-prolonging ART, DALYs are almost entirely captured by the change in mortality; for this analysis we ignore the relatively modest disability component (about 1.5%), but retain the DALY nomenclature for comparability. Costs and DALYs are discounted to 2010 at 3% per year.

Scenarios are compared incrementally to assess net DALYs, net cost or savings, and, for scenarios with a net cost, cost-effectiveness. Thus, net DALYs represent the gain in life years between successive ART scenarios. Net costs (or savings) represent the gain (or reduction) in total costs due to added costs of expanded ART and averted costs from averted HIV infections. With net costs, we calculate the cost per DALY averted. If there are net savings, the cost-effectiveness ratio is misleading and is not reported.

### Sensitivity analyses

We conducted one-way sensitivity analyses on all variables. In general, we used ranges that represent values reported in the scientific literature. For variables that characterize program functioning (e.g., drop out rates), we used values that represent well-run HIV testing campaigns and ART programs. That is, we did not attempt to answer the question, What if the HIV testing or ART effort was substantially non-functional? For variables that represent policy choices, such as ARV drug price schedule or monitoring regimen, we conducted categorical sensitivity analyses, i.e., option A or option B, but no intermediate value. We also used a categorical approach to compare current vs. enhanced prevention, and temporally even vs. acute-phased concentrated HIV transmission.

We conducted multivariate sensitivity analyses for the 34 continuous variables that affect results by more than 0.5% (excluding 12 variables below this level). We created a macro routine in Excel that samples each variable (using a flat statistical distribution), for 1000 iterations per simulation run. Some input value ranges are asymmetrical around the base case value, when we examine only worsening from the best practice assumption in the base case. Thus, we also present the static result that corresponds to placing these input values at the mid-point of their sensitivity analysis ranges. We report the mean and distribution of findings for these simulations.

## Results

**Figure 1:**
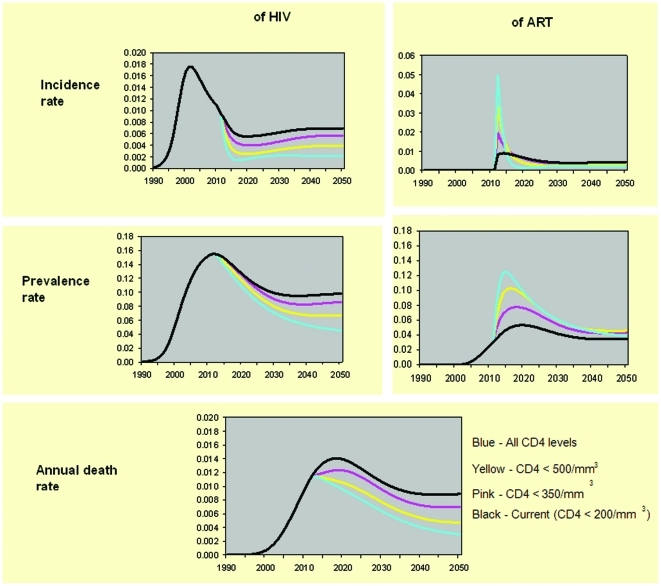
Key epidemic indicators by ART scenario, over time, enhanced prevention scenario to 2050. This figure presents key epidemic results. The adult population is the denominator. “Blue” is all CD4 levels, “Yellow” is <500/mm^3^, “Pink” is <350/mm^3^, and “Black” is current <200/mm^3^. The “Incidence rate” graphs show that the rate of new HIV infections drops most sharply with higher ART use due to more inclusive CD4 criteria. The “Prevalence Rate” graphs show a similar but more gradual decline in HIV prevalence with more ART, and also show that differences in ART use in the short term converge over time due to averted infections The “Annual death rate” graph highlights the benefit of expanded ART on death rates.

**Figure 2:**
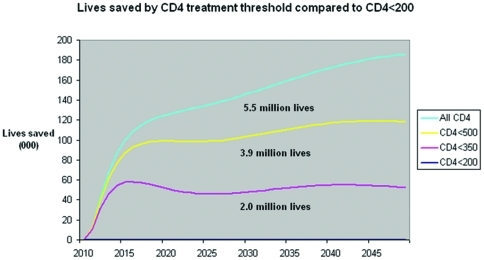
Lives saved by CD4 treatment threshold compared to current CD4<200 baseline. Graph shows lives saved by CD4 treatment threshold compared to current CD4<200 baseline with the <350 scenario portrayed in “pink”, <500 in “yellow” and all CD4 in “blue”. Lives saved increase with earlier access to ART.

**Figure 3:**
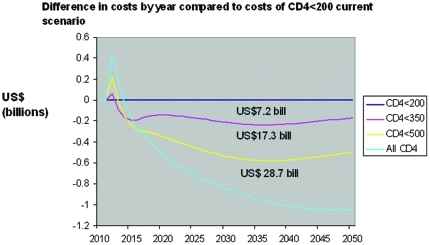
Annual cost by scenario compared to current prevention scenario baseline, 2010–2050. This figure shows the annual cost by ART scenario compared to the projected baseline of <200 current scenario. Totals represent cumulative cost savings over 2010–2050 time period. Cost neutral time points cluster around 2015. Discounted savings over 40 years are 3.9, 8.8, and 13.8 billion for <350, <500, and all CD4 cells, respectively.

**Figure 4:**
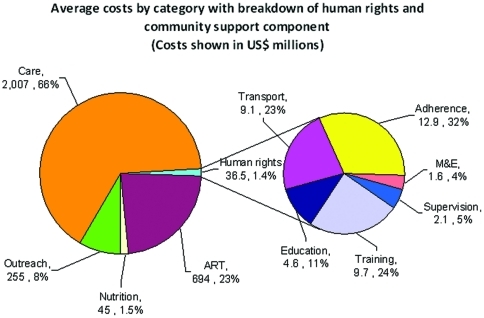
Average annual costs by category with breakdown of human rights and community support components. The pie represents the annual costs based on a CD4<350 scenario without additional prevention averaged over 40 years. Care includes hospital and primary health care (excluding nutrition and human rights), which have their own slices in the pie. Outreach represents the costs of the community-based campaign excluding human rights and community support costs. ART represents ARV costs, aboratory and clinic visits are included in Care category. The other categories are self-explanatory and further details can be found in the Information S1 document.

**Figure 5:**
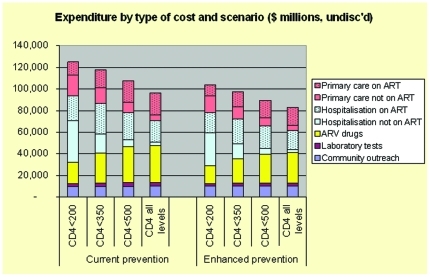
Undiscounted expenditure over 40 years by type of cost and scenario. These histograms show how projected expenditures over 40 years vary as a function of the ART scenario. As ART intensity rises, overall spending drops, with a modest rise in ARV drug costs and a larger drop in hospital costs.

**Figure 6:**
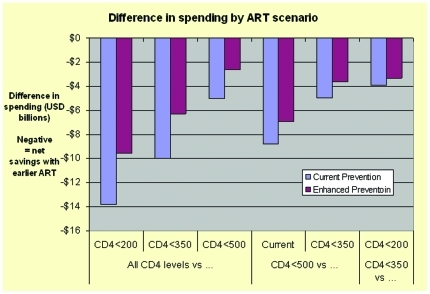
Difference in spending by ART scenario, 2010–2050 (discounted to 2010). This bar chart represents the difference in spending between different ART scenarios over 40 years (discounted to 2010). The ART scenario comparison is indicated on the horizontal axis, and the cost difference on the vertical axis (in millions). Negative numbers indicate fewer costs with more use of ART. Findings under current prevention impact are indicated in blue, and under enhanced prevention in maroon. The differences between scenarios are greater for current than enhanced prevention.

**Figure 7:**
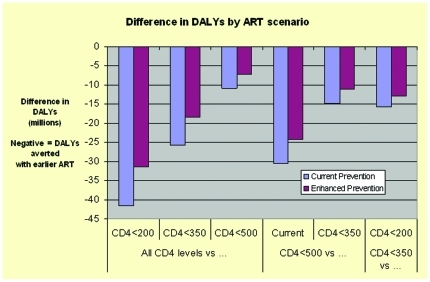
Difference in DALYs by ART scenario, 2010–2050 (discounted to 2010). This bar chart represents the difference in Disability Adjusted Life Years (DALYs) between different ART scenarios, over 40 years (discounted to 2010). The ART scenario comparison is indicated in the horizontal axis label. The DALY difference is indicated on the vertical axis (in thousands). Negative numbers indicate fewer DALYs with more use of ART, representing lower disease burden. Findings with current prevention are indicated in blue, and with enhanced prevention in maroon. The differences between ART scenarios are greater under current prevention impact than assumed enhanced prevention impact.

Results are presented in Tables 2 and 3 and in figures 1, 2, 3, 4, 5, 6, 7 as indicated. Our baseline findings represent the potential impact of best practice HIV testing and ART programs. We also performed sensitivity analyses regarding key assumptions (see SI).

**Table 2:** Summary of results, health and cost effects of ART scenarios with current prevention projected over 5 and 40 years (2011–2050).

Key findings ART scenarios with current prevention projected over 5 and 40 years (2011–2050)									
	ART Scenario								
	CD4<200		CD4<350		CD4<500		All CD4		
	5 yr	40 yr	5 yr	40 yr	5 yr	40 yr	5 yr	40 yr	All CD4 vs CD4<200
Person years on ART (M)	8	79	12	109	17	131	22	134	134
*Change from prior*			*4.3*	*30.4*	*4.7*	*21.7*	*4.6*	*2.8*	*54.9*
New HIV infections (M)	1.6	10.3	1.3	8.7	1.0	6.7	0.6	4.7	5
*Change from prior*			−0.3	−1.6	−0.3	−1.9	−0.4	−2.1	−5.6
Deaths (M)	1.5	12.5	1.3	10.6	1.2	8.7	1.2	7.1	7
*Change from prior*			*−0.2*	*−1.9*	*−0.1*	*−1.9*	*0.0*	*−1.5*	*−5.4*
DALYs due to deaths (M)	21	109	18	93	17	78	17	67	67
*Change from prior*			*−2.7*	*−15.7*	*−1.0*	*−14.8*	*−0.4*	*−10.9*	*−41.4*
Total HIV care costs (M)	14,223	75,339	13,879	71,480	14,056	66,517	14,594	61,505	61,505
*Change from prior*			*−344*	*−3,859*	*177*	*−4,963*	*537*	*−5,012*	*−13,833.9*
Cost per DALY averted, vs. prior			N/A	N/A	$173	N/A	$1,351	N/A	N/A

**Table 3:** Results by ART scenarios with enhanced prevention projected over 5 and 40 years (2011–2050).

Results for ART scenarios with enhanced prevention projected over 5 and 40 years (2011–2050)									
	ART Scenario								
	CD4<200		CD4<350		CD4<500		All CD4		
	5 yr	40 yr	5 yr	40 yr	5 yr	40 yr	5 yr	40 yr	All CD4 vs CD4<200
Person years on ART (millions)	8	66	12	89	17	105	21	110	110
*Change from prior scenario*			*4.2*	*23.5*	*4.6*	*16.1*	*4.4*	*4.4*	*44.0*
New HIV infections (millions)	1.2	7.6	1.0	6.2	0.8	4.7	0.5	3.3	3
*Change from prior scenario*			*−0.2*	*−1.4*	*−0.2*	*−1.5*	*−0.3*	*−1.4*	*−4.3*
Deaths (millions)	10.1	10.4	1.5	8.9	1.3	7.4	1.2	6.5	6
*Change from prior scenario*			*−8.6*	*−1.6*	*−0.2*	*−1.4*	*−0.1*	*−1.0*	*−4.0*
DALYs due to deaths (millions, NPV)	21	95	18	82	17	71	17	63	63
*Change from prior scenario*			*−2.7*	*−13.0*	*−1.0*	*−11.2*	*−0.4*	*−7.2*	*−31.3*
Total HIV care costs (millions, NPV)	14,119	65,120	13,785	61,821	13,969	58,196	14,503	55,559	55,559
*Change from prior scenario*			*−333.7*	*−3,298.6*	*183.9*	*−3,624.6*	*533.7*	*−2,637.0*	*−9,560.2*
Cost per DALY averted, vs. prior scenario			N/A	N/A	$182	N/A	$1,381	N/A	N/A

**Notes:** ART scenarios refer to the CD4 level at which ART is offered. Enhanced prevention assumes a 40% reduction in HIV incidence before expansion of ART. See text for detail.

### ART use

Person-years on ART varies from 79 million with “current” to 109 million with “<350 cells/mm^3^”, 131 million with <500 cells/mm^3^, and 134 million with all CD4 levels (about 83% of HIV-infected), over 40 years (Tables 2 and 3). This pattern reflects expanded ART initiation at higher CD4 counts which results in more averted HIV infections and a reduction in future demand. With enhanced combination prevention (40% lower incidence), ART use varies from 66 to 110 million person years, reflecting fewer new HIV infections.

The incidence and prevalence of ART use by year is shown in Figure 1, for enhanced prevention. The initial prevalence reflects the CD4 starting point in each scenario. Over time ART use drops in the more inclusive scenarios, due to averted HIV infections. The “all CD4 level” scenario has the second lowest ART use by 2050.

### Health outcomes

New HIV infections over 5 years decline by 265,000 (17%) between “current” and “<350 cells/mm^3^” (Table 2). Over 40 years the decline is 15%, from 8.7 million (“current”) to 7.3 million (“<350”) new infections (Table 3). When compared with the current scenario, expanding from current to <500 prevents an additional 585,000 and 3 million new HIV infections over 5 and 40 years, respectively. “All CD4 levels” when compared with “current” scenario prevents 947,000 and 4.7 million over 5 and 40 years, respectively. The comparable values for enhanced prevention are approximately 30% lower in each ART access scenario. HIV prevalence rises slightly over the first 3–5 years of scaling-up ART due to decreased mortality, and then drops as a result of reduced HIV incidence, in proportion to the intensity of ART use. Differences grow over time for both prevalence and incidence (Figure 1).

Deaths over 5 years decrease from 1.5 million for “current” ART to 1.3 for <350 cells/mm^3^, 1.23 for <500 cells/mm^3^ and 1.20 million for “all CD4 levels” under current prevention (Table 2). Over 40 years, deaths decrease from 12.5 million for “current” ART to 10.6 for ≤350 cells/mm^3^, 8.7 for <500 cells/mm^3^ and 7.1 million for “all CD4 levels” under current prevention, and 10.4, 8.9, 7.4, and 6.5 million respectively for enhanced prevention (Table 3). Differences between scenarios start within two years due to the slowing of disease progression, and expand later with the delayed mortality benefits of averted HIV infections (Figure 1). Deaths over 5 years decrease from 1.50 million for “current” ART to 1.30 million for <350 cells/mm^3^, 1.23 million for <500 cells/mm^3^ and 1.20 million for “all CD4 levels” under “current prevention” (Figure 2). The values are nearly identical for “enhanced prevention”, since mortality prevention benefits in the short term derive from earlier treatment of already HIV-infected individuals. Although epidemic elimination (defined as annual HIV incidence rate <0.001) is not achieved using the assumption of 92% reduction in transmission on ART, it can be achieved with various modifications of base case assumptions including 97% reduction in transmission while on ART with “enhanced prevention”.

### Cost-effectiveness: costs and health impact

When compared with the baseline “current scenario”, the cumulative undiscounted cost savings are 7.2, 17.3, and 28.7 billion dollars for <350, <500 and all CD4 count scenarios over the 40 year period (Figure 3). Break even in cumulative costs is achieved around 2015. Compared with “current”, the “<350 cells/mm^3^” scenario has discounted costs that are lower by $504 million over 5 years, and $3.9 billion over 40 years. With enhanced prevention, “<350 cells/mm^3^” reduces costs by $3.3 billion versus “current” over 40 years, “<500” by $3.6 billion more, and “all CD4 levels” by a final $2.6 billion (Table 2 and 3, Figure 3).

Annual costs rise for more intensive ART use for several years, and by around 2017 are below the less intensive ART scenarios, with differences growing over time (Figure 3). The “<350 cells/mm^3^” strategy has only a small and brief annual cost rise over “All CD4 levels” because of an immediate drop in inpatient utilization and costs that nearly balances the increase in ART and outpatient costs. Differences in cumulative costs grow steadily over time (data not shown). The average annual costs by category reflect the relatively small proportion of costs due to ARV drugs (Figure 4) and the shift in type of expenditures reflects the addition of ARV drugs and a drop in hospital costs (Figure 5). Although the greatest differences in spending are reflected when contrasting immediate and current scenarios, savings are accrued for all scenarios when contrasted with the current approach (Figure 6).

DALYs decline from 109 to 67 million, discounted, with an 11 to 16 million difference between each ART scenario. Enhancing prevention (40% lower HIV incidence) reduces DALYs averted by expanding ART by 27% and cost differences by 37% (Tables 2 and 3, Figure 7). With enhanced prevention, these differences drop by about one quarter (Tables 2 and 3, Figure 7). The proportionate cost difference across scenarios is smaller than for DALYs because ART only improves health (through treatment and prevention) but has competing effects on costs (increased in the short term, decreased in the long term).

### Sensitivity analyses

Varying most input values over specified uncertainty ranges preserves the qualitative findings of net savings and substantial averted DALYs (see SI section 5).

#### One-way

A higher annual drop-out rate on ART (3% or 5%, instead of the 1.5% base case value from the Malawi national program) results in a decrease from 41 million DALYs averted over 40 years in the base case to 33–37 million DALYs averted for the higher drop out rates. The decrease is consistent across the CD4 levels, since more time in ART always decreases DALYs. The cost reduction drops from $14 billion in the base case to $13 billion at 5% drop-out. The effect varies by CD4 level, due to competing influences on costs as individuals exit ART. The initial refusal rate (4 to 12% range) makes a difference of <500,000 DALYs averted over 40 years, mainly due to repeated ART offers to refusers (not in table).

Life expectancy off ART has opposite effects on DALYs and costs: an increase to 14 years decreases DALYs averted but increases cost savings with ART. The increase in life expectancy from ART (to 3.5 fold from 2 fold) increases both DALYs averted and savings.

If ART reduces transmission by 99% instead of 92%, net savings from all CD4 level versus current reach $17.5 billion. If ART reduces transmission by only 85%, the savings are $12.6 billion. The scenarios differ in terms of DALYs only modestly for two reasons: many DALYs averted derive from the current benefits of treatment not prevention, and a difference of 1% vs. 15% in residual transmission risk among individuals on ART yields a smaller relative difference when adjusted for the proportion not on ART (84% in “all CD4” and greater in other scenarios).

Higher 2009 South Africa ART costs results in *all CD4 level* ART saving $0.6 billion versus *current ART*. However, intermediate scenarios have net higher costs of $3.05 and $0.14 billion versus less ART-intensive scenarios, with cost-effectiveness of $9–194 per DALY averted. Using broadly higher costs based on South Africa data (higher ART and monitoring costs, longer inpatient stays and higher daily costs, compared to our baseline values) results in higher net savings than the base case.

The rate of ART regimen change is inversely proportional to net savings, with no effect on DALYs in our model. An unfavorable case that combines full South Africa costs with low testing coverage, high ART refusals, and high drop outs leads to 42% fewer DALYs averted and 30% lower net savings, with a cost of $59 per DALY for the increment from CD4 count >500 cells/mm^3^ to “All CD4 levels”. Use of cotrimoxazole in the CD4 level one higher than designated for initiating ART results in 3–15% reduction in DALYs averted and small changes in net savings that vary according to the comparison. Testing every 3 years instead of annually results in 14% more DALYs overall (i.e., higher disease burden), and 5–47% smaller differences between ART scenarios. This analysis conservatively assumes CD4 testing on the same frequency.

The reduction in inpatient days (from 7.1 days per year off ART to 1.7 on ART, on average) is unrelated to DALYs, and directly related to savings. Inpatient day cost has a similar effect.

We examined implementing training in integrated management of HIV, as recommended by WHO (not in table). A 5-year program training 50% of PHC staff, followed by annual turnover at 10%, would cost $156 million in the first 5 years and $115 million per five year period thereafter, for a discounted cost over 40 years of $569 million, or about 7% of net savings. We ignore likely compensatory savings from decentralization to less expensive facilities and better organization of care.

#### Multivariate

Some input value ranges are asymmetrical around the base case value, when we examine only worsening from the best practice assumption in the base case (See SI Section 5). Thus, as a comparison with the multivariate simulations, Table 4 in the Information S1 document contains a static result that corresponds to placing these input values at the mid-point of their sensitivity analysis ranges. DALYs averted are 38,000, compared with 41,000 in the base case. Net savings are $10.5 billion, compared with $13.8 billion in the base case.

The “even stages” analysis assumes that HIV risk is not concentrated in the acute phase, similar to our base case assumption. The mean change in DALYs and costs is similar to the static case described immediately above. The distribution of findings is shown in the SI Section 5. Net savings are achieved in 99% of simulations. With 30% of HIV transmission in the 3 month acute phase, DALYs averted drop by 26% to 31 million and the net savings drop by 7% to $12.8 billion.

## Discussion

As part of a well-run ART program, our projections suggest that a ‘front loaded’ investment into expanding ART for people living with HIV with a CD4 count <350 cells/mm^3^ could yield results almost immediately. Expanded access could reduce new HIV infections by an estimated 265,000 over five years and 1.4 million over 40 years. Over the near term, the investment strategy could save lives by reducing estimated deaths by 200,000 with a projected savings of $504 million over 5 years. Over 40 years, it could reduce estimated deaths by 2.9 million, disability adjusted life years by 15.7 million, and an estimated reduction in costs of $3.9 billion. These projections support WHO's recent recommendations to start ART earlier for those with ≤350 CD4 cells/mm^3^ and South Africa's recent decision to expand access [38].

There is increasing evidence that earlier treatment may be advantageous for both prevention and clinical care [14]. However, starting earlier also comes with potential risks including potential adverse effects and difficulties accessing second line treatment. Modeling the health and economic impact provides an opportunity to examine a number of interventions and scenarios. Expanding to CD4 count <500 cells/mm^3^could further reduce estimated deaths by 1.9 million and disability adjusted life years by 14.8 million over 40 years. This expanded access strategy could decrease costs by an estimated $100 million over five years and $5.0 billion over 40 years. Although we based our assumptions on a well-run program, the result of net savings is robust to uncertainty in most input assumptions, such as transmission suppression, reduction in inpatient days, and use of pre-ART cotrimoxazole prophylaxis. However, savings depend substantially on South Africa using lower cost international sourcing for anti-retroviral drugs. Most people in South Africa are starting ART very late in the course of disease, however, extending ART access to people earlier after HIV infection has potential additional economic and epidemiologic impact. According to our projections, adopting a policy of ART for all CD4 levels could result in a further estimated decline of 3.3 million infections, 3.5 million deaths, 25.7 million DALYs, and $10 billion over 40 years, as compared with CD4 count <350 cells/mm^3^. The expansion to all CD4 levels could achieve cost breakeven by 2022 (within 10 years) versus “current”. In the context of enhanced prevention (40% lower incidence), ART expansion could still substantially reduce HIV health burden and lower costs, and could be an essential contributor to epidemic elimination by the standard of annual HIV incidence <0.001.

The substantial projected net savings reflect the anticipated epidemic impact of ART, as well as economic and epidemic conditions in South Africa. Over multiple years and decades, the assumed 92% reduction in HIV transmission among those on ART sharply reduces new infections and associated medical costs. These savings are substantial per person due to the relatively high cost of medical care in South Africa, as compared with most of sub-Saharan Africa [58]. Indeed, we found that an expensive intervention like ART yields savings even in the short term (5 years), based on slowed progression to more expensive disease stages and data suggesting that individuals with CD4 counts at least as high as 500 have serious HIV-related morbidity and mortality and associated inpatient care, which ART reduces by 60–80% [59], [60], [61], [62]. The large overall magnitude of savings reflects the large scale of the HIV epidemic in South Africa. Past cost-effectiveness analyses of ART in Africa, including our own, omitted the indirect, dynamic epidemic effects of ART through reduced HIV transmission and were conducted in settings with lower medical care costs. Thus, they found substantial net costs and relatively high cost-effectiveness: $597 per DALY averted in Uganda, including prevention effects but only in immediate partners; and $620 per DALY averted in Cote d'Ivoire [59], [63]. An analysis of future resource needs for AIDS in low- and middle-income countries predicted 20% savings with enhanced targeted prevention and 44% higher costs with broad program scale-up, but did not consider the HIV infections and costs averted with expanded ART [41]. A cost-effectiveness analysis of ART expansion in Vancouver, Canada, using a willingness-to-pay approach, estimated a net benefit of US$ 900 million over 30 years [64].

Our analysis links within one framework three key issues in global HIV: low but growing knowledge of HIV status, falling ART costs, and evidence for ART suppression of HIV transmission [4] [34] [65], [66]. According to the WHO, between 2005 and 2008, the median percent of persons living with HIV who reported receiving HIV test results increased from 15% to 39% [67]. Still, in some settings the need for increased HIV testing is stark; the Kenya AIDS Indicator Study found that in 2007 only 17% of those testing HIV-positive knew of their status [68]. Second, the cost of ART is falling, in PEPFAR to the range of $400–500 per person year, including about 39% for the ARV drugs; there remain issues in fully understanding costs and their determinants [69] [70]. Finally, quantitative evidence of suppression of HIV transmission during ART is converging on an over 90% reduction, although this remains to be confirmed during field implementation in larger-scale programs and over a longer-term than the recent cohort studies and HPTN 052 clinical trial which reported a 96% reduction [14] [22], [27]. Importantly, initial evidence suggests that viral load suppression by ART is accompanied by lower risk behaviour, in South Africa [71] and elsewhere [72]. Other recent analyses have costed and projected a significant combined prevention impact of expanding ART for the majority of people living with HIV with a CD4 cell count <350 [34]. In Botswana, patterns in sexual behaviour have remained relatively stable since 2000 and the country scaled up access to treatment from less than 5% in 2000 to over 80% which it has maintained since 2009. The annual number of new HIV infections has declined by over two thirds since the late nineties and data suggests that the number of new HIV infections in Botswana is 30% to 50% lower today than it would have been in the absence of antiretroviral therapy [3]. Recent studies show that treatment can be up to 96% effective in preventing HIV transmission among couples [14]. Taken together, these factors suggest large need and opportunity for expanded testing and earlier treatment.

Our study has important limitations. We do not explicitly portray anti-retroviral resistance. Resistance has been proposed as major drawback of expanded access to ART in modeled analyses [33]. However, accumulating empirical evidence suggests that these concerns are not borne out with ART in community practice, where little clinical relevant resistance has been documented (e.g., Vancouver [73]), perhaps due to the recent advances in ART that increase adherence and decrease resistance [74]. We do capture the effect of expected modest resistance through the shift to (more expensive) second line ART. We assume no exhaustion of ARV regimen options. More generally, our model has only one mechanism whereby earlier ART initiation can increase mortality: ART drop-out rate. HPTN 052 demonstrated a 40% reduction in health related events for those started immediately below 550 CD4 count and current clinical trials such as START and TEMPRANO will provide additional data on clinical outcomes with early ART initiation [14]. Our epidemiologic model is not risk, age- or gender-structured, so we could not examine targeted strategies. We are adapting the model for this purpose. We assume excellent ART program operation, based on the example of Malawi, one of the poorest countries in the world. Although we explore the implications of weaker programming, such as higher drop-out rates but do not examine what would happen in the face of lower levels of access to HIV testing and counseling and/or linkage to care and treatment. However, our analysis should be considered to represent the potential gains from the expanded use of ART in programs with optimal programmatic achievements that can be found in some of the best programs serving individuals under current treatment guidelines (lower CD4 levels). Given the recent recognition of the short and long-term mortality benefits of ART even at high CD4, we think this perspective is reasonable. Furthermore, we believe that a “best practices” approach is consonant with increasing emphasis on program performance in global health. Donors are investing more cautiously, driven by global economic problems and tighter budgets. Thus, programs must show good results in order to continue receiving broad support; ineffective programs must be improved or their funding redirected to programs with strong performance. Ultimately, the objective of donor initiatives should be to ensure that money is spent wisely and leads efficiently to better health for the world's poor. A best practices approach sets the bar high, as we think is appropriate as programs increasingly focus on expanding access to earlier HIV testing, counseling and ART with accompanying monitoring and evaluation of implementation.

Decisions around the cost of end-of life care are important and we assumed that end-of-life care is less expensive with ART, i.e., that there is no final intensive utilization comparable to that observed with no ART. Although, this assumption has important policy and cost implications, for our analysis of early ART initiation, it has little consequence: individuals with CD4 count <200 cells/mm^3^ are offered ART in all scenarios. However, if the assumption leads to understating lifetime costs of HIV care, then we underestimate the savings due to averted HIV infections. Our results are conservative in that we also omitted several important types of health benefits and care savings: preventing tuberculosis in index patients and contacts [75], benefits to family members [60], and benefits above CD4 count of 500 cells/mm^3^ [62]. Additionally, by using annual CD4 testing for HIV-infected not yet on ART, we likely delay ART initiation compared with every 6 month testing often used in operating programs. We also exclude the broad economic and social benefits of reduced HIV infections which are likely to be very substantial, but uncertain in magnitude and beyond our scope [76].

For the cost analysis, we assume that the savings due to averted inpatient care can be captured in financial savings or in expanded services for other health conditions. This assumption, though potentially difficult to operationalize in the short term due to fixed and under-utilized health care resources, is the same as in other cost-effectiveness and net cost analyses. Our findings apply only to South Africa, whereas the economics of expanded ART may differ in settings with different cost structure or epidemiology [76]. For example, due to South Africa's relatively high cost of inpatient care, the cost savings from reduced hospitalizations for persons with HIV are larger than in most other African countries, while the cost of ARV drugs is comparable. Lower HIV prevalence may lead to a higher relative cost burden from HCT, though this distinction largely disappears after the first round of testing. Recognizing this geographical limitation, we plan to use our model to assess other settings with a set of revised input values.

Other ‘ART for prevention’ evaluations in recent years have been less optimistic than this one. As noted above, a theoretical analysis for San Francisco found a substantial risk of epidemics of resistant ARV strains with scale-up [33]; empirical evidence to date fails to confirm these risks [73]. Another modeling analysis found that a “test-and-treat” strategy can substantially reduce HIV transmission, “broadly confirm[ing] the main findings” from our prior model [77]. However, the analysis also found that the intervention works less well in populations with highly heterogenous risk segmentation (i.e., high risk core groups and non-sustaining risk in low risk remainder), or if coverage is less than 50%. It found that for the cost-efficiency ratio [reduction in HIV incidence] divided by [cost for ART and HCT], 80% coverage was optimal along with a testing interval of 1–5 years depending on the epidemic scenario. Importantly, the analysis did not incorporate ART drop-out and offsetting savings in medical care as in this paper. Greater ART drop-out rates substantially decrease the expected scale of health benefits, with less pronounced effects on net savings. Some projections place more emphasis on data from cohort studies which suggest a substantial concentration of HIV transmission in the acute phase. Although the surveillance and other evidence from South Africa demonstrates a lower contribution, accounting for a larger proportion of transmission in the acute phase would only lower DALYs averted by one-quarter and savings by 7%. Perhaps most importantly, the 92–96% reduction in HIV transmission during ART as found in recent cohort studies and a clinical trials, and the patient retention and adherence rates taken from Malawi's best-practice program, remain to confirmed empirically in the context of other large-scale programs, sustained over a longer-term as needed in South Africa.

WHO recommends expanding access to ART for ≤350 cells/mm^3^ and recognizes that expanding access to ART within a human rights framework is paramount: without community engagement and support expanding access to ≤350 cells/mm^3^ will likely be impossible. It is critical that there is community engagement in the planning and implementation of the program, that there is no coercion to be HIV tested or take ART, and that there is adequate support for individuals with questions and concerns. Contrary to many other similar studies, we included the cost of ensuring many components of a strong human rights framework [78]. A related issue is the motivation for individuals with early HIV disease to accept ART, given the low risk of HIV-related death. How early to start ART remains controversial, however, despite the growing scientific evidence and potential for individual [62], public health and economic benefits, several reservations have been raised regarding the desirability and feasibility of expansion to the CD4 count <500 cells/mm^3^ and “all CD4 levels” [33]. The 2009 WHO international expert committee, after a rigorous review of the scientific evidence base, feasibility, and cost considerations, reached consensus for starting ART at <350 cells/mm^3^ [38].

On the practical side, a major concern has been the logistical challenge of expanding care, which seems at first glance would disrupt the health system. We agree that expanding ART to CD4 count ≤350 cells/mm^3^ levels will be, in many settings, a difficult undertaking; even the resource needs to maintain current efforts in the intermediate term are substantial [79]. However, we found that for South Africa, in cost terms, intervening earlier is predicted to pay for itself within 4 to 12 years (with gains accumulating over time as infections are averted), so expanding ART is initially less a jump in resources than a shift from inpatient to ambulatory care. The costing assumes more use of lay providers and task shifting to manage the increase in routine management of care, leaving the existing clinical staff to deliver clinical care. Regarding HIV control strategy, some experts argue that effective, efficient traditional HIV prevention should be scaled up. We agree, and point in particular to interventions in high risk groups such as sex workers, male circumcision, and HIV testing and counseling, especially in discordant couples [80], [81]. Indeed, our model found that epidemic elimination is most likely with the combination of these strategies and ART expanded to all CD4 levels. Finally, we recognize the link between program performance and impact. In order to yield broad epidemic benefits, an ART for prevention strategy must achieve high HIV testing levels, high acceptance of ART, and high retention and adherence within ART, especially on first line regimens. Thus effective implementation must promote, monitor, and achieve high performance in these areas.

Our analysis offers several insights into the economics and impact of ART for prevention in South Africa. First, moving from current ART practice to WHO's recommended starting rule of CD4 count ≤350 cells/mm^3^ is likely to save substantial lives and money, within a few years and more over time. South Africa recently adopted this CD4 level as the guideline for pregnant women and TB patients and we hope accumulating data will allow us to refine our analyses to include these groups. Second, extension of ART to CD4 count <500 cells/mm^3^ and all CD4 levels is likely to save even more lives and money, albeit requiring large ‘front loaded’ investments that may be challenging to marshal. Third, these favorable findings owe much to the potent combination of high averted inpatient costs, low antiretroviral drug costs, and many HIV infections averted. Finally, the most profound epidemic abatement can occur only in the context of enhanced traditional prevention. We believe that economic analysis is helpful to understand the broad implications of a complex intervention like ART for prevention. For example, only through integration of diverse data on health care utilization and costs were we able to identify the favorable economics of expanded ART in South Africa. From a resource mobilization perspective, our findings suggest that through a ‘front loaded’ investment strategy the costs of responding to HIV epidemic could reach a level requiring only minimal external support. Similar economic modeling will be valuable in this and other geographic settings, to increase the robustness and subtlety of our understanding of ART for prevention. The models should also allow modification of parameter values to explore policy options under consideration by health officials. In conclusion, we believe that accumulated evidence and analyses support the implementation of phased program implementation to assess the capacity of health systems to deliver and sustain earlier ART, including HIV testing and counseling, ART uptake, retention and adherence, and its community-level impact on HIV transmission and costs.

## Supporting Information

Information S1 Supporting information for this manuscript can be found in the supporting information document which is designated as “Information S1”
(DOC)
