# Supplementary material for: Expanding ART for Treatment and Prevention of HIV in South Africa: Estimated Cost and Cost-Effectiveness 2011-2050
Source: PLoS One. 2012 Feb 13;7(2):e30216. doi: 10.1371/journal.pone.0030216 (PMC3278413; doi:10.1371/journal.pone.0030216)
Supplement: Information S1 — Supporting information for this manuscript can be found in the supporting information document which is designated as “Information S1” (DOC) [file pone.0030216.s001.doc]

# Expanding ART for treatment and prevention of HIV in South Africa: cost and cost-effectiveness 2011-2050

# 1.0 Introduction

Using epidemiological projections of the epidemic of HIV in South Africa and the estimated impact of providing HIV-positive people with anti-retroviral therapy under various scenarios we estimate the economic costs and benefits of this intervention. We draw on detailed, district level costs, aggregated nationally. The costing framework is discussed briefly in the paper to which this supporting information refers and in detail below.

# 2.0 The economic model

The epidemiological model, which provides the basis for the economic analysis, is described in detail elsewhere.[^1^](#_ENREF_1) The model includes four stages of HIV-infection corresponding loosely to the four stages of HIV-infection as defined by the World Health Organization. The most important parameters are the frequency with which people are tested for HV, the CD4^+^ cell count at which people start treatment, the coverage of testing and treatment, the drop-out rate for those that start treatment, the extent to which other interventions or changes in behaviour reduce transmission. For full details the reader is referred to the original publication and supporting information. The model is not structured by age or sex.

The burden of HIV in each province, based on the provincial population and prevalence of HIV, was used to determine the costs and benefits in each province and aggregated to give overall, national figures. Costs are in 2009 US$, local prices are converted at the average exchange rate for 2009, historic prices are inflated at 4.9 % p.a., future costs are discounted at 3.5% p.a.

We consider costs incurred by the public health system including clinic visits, staff time, hospitalisation, and testing and treating people living with HIV. We assume that the scale-up of anti-retroviral therapy (ART) will be through existing structures but where additional resources are needed their costs are included.

Significantly expanding the access to ART will require substantial community involvement and support, especially in remote rural areas and these might include door to door and public health campaigns.[^2^](#_ENREF_2) We base the cost of community testing on a public health campaign in Western Kenya in which 47,000 adults, making up 80% of the adult population, were tested for HIV and provided with insecticide impregnated bed-nets and filtered drinking straws in seven days.[^3^](#_ENREF_3) Costs incurred in the campaign were used to estimate the cost of reaching institutionalised populations, such as prisons or the military, or major parastatal or public sector employers.

Some of the costs of ensuring that people’s rights were respected were included in the Kenya campaign and used in this exercise but additional costs were included to ensure that the design, management and monitoring of the expanded programme would be fully supportive of the rights of individuals.

High levels of adherence to drug-regimens are essential if expanded provision of ART is to have a significant impact. We include the cost of providing support programmes in each community to monitor and counsel patients on the importance of good compliance as well as the cost of providing nutritional support for undernourished patients.

The cost of providing universal access to ART for everyone will run into billions of US$ per year. To minimize costs while creating jobs and stimulating local economies and there will be considerable economic and social benefit from using, involving and relying on local people to carry out as much of the work as possible.[^4^](#_ENREF_4) Having trained such people to provide support, counselling and advice to people infected with HIV they should also be engaged in helping to deal with other health care issues of importance most notably tuberculosis but also childhood illnesses, nutrition and malaria where it is present.

# 3.0 Costing categories and assumptions

## 3.1 Health care utilization

Clinical work is assumed to take place in the current health system without the need to modify or develop additional facilities and has been benchmarked to reflect the level of health-service provision in South Africa. Costs, resources, infrastructure and the scale of the HIV epidemic, differ among countries and will require separate estimates.

Data on the use of health services was taken from recent studies reported in a study in Uganda.[^5^](#_ENREF_5) The median number of out-patient visits per year increased from 5.5, before starting ART, to 9.5 after starting ART while the median number of in-patient days per year decreased from 7.1 before starting ART to 1.7 after starting ART. The out-patient visits and in-patient days were distributed among the four stages of HIV infection.[^6^](#_ENREF_6) Under these assumptions HIV-related conditions account for 44% of all hospital beds and 28% of people accessing primary health care in South Africa.

In-patient care was allocated separately to Level 1 (general), Level 2 (specialized) and Level 3 (tertiary) hospitals using pre-ART data[^6^](#_ENREF_6) and the same distribution was assumed for those with HIV. The costs of one in-patient day, one out-patient visit and one primary health care visit as given for South Africa by the World Health Organization were within 4% of the costs estimated from the total expenditure summed over all provinces. To this we added the facility costs including treatment of opportunistic infections, the costs of ART and the associated laboratory costs (Table 1).

Table 1. Bed-day and out-patient costs and primary health care coverage and costs per visit for the three levels of hospital.

| Care level | 1 | 2 | 3 |
| --- | --- | --- | --- |
| Bed-day cost (US$) | 80 | 105 | 143 |
| Out-patient visit (US$) | 28 | 39 | 58 |
| Primary health care coverage (%) | 50 | 80 | 95 |
| Primary health care visit (US$) | 12 | 14 | 21 |

For comparison, a recent HIV cost projection analysis[^7^](#_ENREF_7) for South Africa reached a similar estimate of the cost for a non-ART outpatient visit (US$28) but a higher cost for an in-patient day ($164). Higher in-patient costs would favour expanded ART by averting the higher medical costs.

In a recent study the costs of inpatient stays were higher for adults infected with HIV than in those not infected with HIV but the difference was mainly due to length of stay rather than cost per day.[^8^](#_ENREF_8) In tertiary hospitals, the cost per in-patient day averaged US$130 for HIV-positive and US$120 for HIV-negative adults in Level 1 hospitals.

In each of the primary health care categories, treatment by nurses or doctors was considered separately for patient on and off ART. Nursing staff were costed at the rates for primary health care and doctors at the rates for out-patients. The increase in the use of health services and associated costs with more advanced disease is consistent with a review of international studies, although data are focused on low CD4 levels.[^9^](#_ENREF_9)

Table 2. Patient visits and the proportion attended to by doctors and nurses.

|  | Pre-ART | | ART | | |
| --- | --- | --- | --- | --- | --- |
|  | Adverse events | TB | Adverse events | TB | ART |
| Visits/patient/year | 4.3 | 12 | 1.4 | 4.3 | 8 |
| Seen by nurse (%) | 67 | 50 | 86% | 70% | 86% |
| Seen by doctor (%) | 33 | 50 | 14% | 30% | 14% |

## 3.2 Anti-retroviral therapy, other drugs, and tests

The costs of first and second line ART drugs were taken from the USAID AIDSTAR-One summary for guidelines for South Africa[^10^](#_ENREF_10) and the AIDS Medicines and Diagnostic Database.[^11^](#_ENREF_11) The first line regimen was tenofovir, lamivudine and nevirapine, with the option to replace tenofovir with zidovudine or stavudine and nevirapine with efavirenz. The second line regimen was zidovudine, didanosine and lopinavir with the option to replace the first two drugs with tenofovir and lamivudine. The estimated costs of the various regimens were as given in Table 3. Starting from a current baseline with 4% of patients on second line ART in 2008 we assume that a further 3% would convert from first to second line every year.

Table 3. Drug costs (US$) for the various drug regimens in South Africa.

|  | 1^st^ Line | 2^nd^ Line |
| --- | --- | --- |
| SMCS | 188 | 595 |
| High | 526 | 1167 |
| Average | 462 | 1077 |
| Low | 259 | 935 |

The use of cotrimoxozole in any stage, with or without ART, was included at cost of US$ 7.20 per patient per year based on the AMDS database unit price and reduced hospitalisation by 69% and progression by 40%.[^4^](#_ENREF_4)

Routine laboratory test costs for monitoring patients on ART were taken from available data per South Africa standards of care. Tests were applied to each of the first and second line ART regimens. For the baseline, we assumed the least expensive monitoring regimen, which is consistent with ART clinical guidelines. The cost of testing for those on first line drugs was US$128 in the first year and US$106 in subsequent years and for those on second line drugs US$62 in the first year and US$31 in subsequent years. The cost of viral load testing for those on first line drugs was US$128 in the first year and US$106 in subsequent years. Testing in South Africa is managed through an independent national laboratory service, the NHLS, no additional component was added for capital and personnel implications of the significant increase in testing rates.

The costs of the community-based campaign were separated into those that were a) planning and management for each province; b) Programme management for each district; c) operational costs for each site; d) Individual costs for each person being tested and counselled.

## 3.3 HIV testing

The number of campaign sites in each of the provinces is based on the population in each district assuming that no more than 7% of the population would have to travel further than 6 km to attend the campaign site. The campaigns are carried out annually but coverage falls at 1.4% per annum after the prevalence peaks in 2021.

The cost of treatment provided in the private sector, especially the mining industry, is borne by the private sector but targeted campaigns to cover prisons and military are allocated to the public sector. Studies of private sector initiatives show similar rates of HIV prevalence to those in the general population, so no adjustment was made to unit costs.

Provider-initiated testing and counselling (PITC) is facility-based and scaled up over time to reach a maximum of 20% of the target (susceptible) population annually. The marginal (additional) costs of PITC were derived from the data regarding the cost of the campaign and included counsellors, testing equipment and a package of 60 condoms per person tested.

High risk groups are accessed through targeted campaigns in clubs, bars or meeting points and/or in institutions. Costs related to setting up sites and localised advertising were excluded. They were also considered to be part of the broader campaign and the organisation and management were subsumed within those costs.

In the model 10% of the population reached in the overall programme would be accessed through the above targeted campaigns, and that these targeted projects would take place in 290 locations.

## 3.4 Human rights and community support

We assume that human rights organisations will be closely involved in the content and oversight of the campaign. An annual budget of US$ 57 k is allocated for the development of human rights materials. US$ 150,000 per year is allocated for consultants to ensure independent verification of the HR activities described below and to audit practices in health care settings. This is supported by an independent exit survey at every site.

One full-time equivalent human rights and community support supervisor per province will support and monitor the human rights and community support aspects of the rollout and implementation of the campaign and facility-based programme in the district and liaise with civil society informally and through monthly meetings. The supervisors are paid local consultant rates at US$ 12 k p.a. One full-time equivalent human rights coordinator per province will be responsible for setting up training courses for the counsellors. The coordinators are paid local consultant rates at US$ 12 k p.a. A human rights and community support advisor will be available for the duration of each campaign and is costed at US$ 60 per day.

People living with HIV support groups are requested to identify a lawyer in each province to be retained by the human rights organisations, to be accessed through a ‘warm line’ paging system. Should legal representation be needed then a one time back-up fund of $100,000 will be established with the majority of expenses expected to be *pro bono* or covered by $25,000 retainer.

Community-based adherence support for people on ART is provided through identified carers in each community provided with a stipend equivalent to USD 120 per month and one supervisor per community paid US$ 2,667 p.a. They would be supported by an electronic mobile phone based system reporting adherence. The cost of this system is US$ 10 per patient year.

Additional support is also provided in the form of a nutritional allowance for undernourished patients, equivalent to US$ 333 per patient year or ZAR 250 per month. Those with a body mass index below < 18 kg/m^2^ will be eligible and we assume that this will include between 5% and 20% of patients.

## 3.5 Costs of training in integrated management

We include a training program for 50% of PHC staff followed by annual turnover at 10% and all receive the full range of appropriate courses recommended by WHO. Costs include start up development of adaptation of WHO guidelines and courses, training trainers and expert patient trainers, costs of transport and per diem for all trainers and trainees. We also include the costs of printing and distribution sufficient quantities of all of the records, charts and registers to every primary health care facility. The costs are not treatment dependent, so are applied equally to all scenarios. The aggregated costs in the 5 yr scale up period are US$156 million, reducing to US$23 million thereafter.

We do not include compensatory savings from two sources: decentralization to less expensive facilities, and better organization of care. The likely impact of decentralizing HIV treatment to primary health centres, with lower unit costs, would be to reduce costs by US$950 million to US$1.26 billion per annum. There would also be improvements in clinical capacity through the training of lay providers to pick up routine non-clinical tasks, freeing clinical staff to see more patients and/or improve quality of care.

## 3.6 Intervention scenarios

We considered four different scenarios with people starting ART when their CD4^+^ cell count falls below 200, 350 or 500/μL or if they start as soon as they are found to have HIV. Full implementation is achieved in 2015.

# 4.0 ARV regimen switching rate

We estimate the rate of switching from first to second line ARV regimen at 3% (1.5-4.5%) p.a.[^12-14^](#_ENREF_12)

# 5.0 Sensitivity analyses for SI

After selecting the baseline values for each of the adjustable variables they were varied either to explore different assumptions or were simply increased and decreased by 50% as indicated in Table 4. The table only records those variables for which the change in the parameter led to a change in the outcome of greater than 5%.

# 5.1 Overall uncertainty analysis

To determine the overall uncertainty in the results all of the parameters were randomly selected from a uniform distribution between the lower and upper limits and the results were calculated for each of the 4 ART treatment thresholds. These results are illustrated in Figure 1 where the net cost savings over 40 years are US$ 2.3 (0.3 to 4.8) billion and the reduction is significant (*p* < 0.05).

Figure 1**.** Distribution of results from multivariate sensitivity analysis.

Table 4. Parameters were varied about their baseline values as indicated in the table and the absolute and relative change in the number of DALYs saved and in the total cost, each over the next 40 years, are given.

| Parameter and | Adjusted values | | ART threshold | Deaths (000) | DALY 40yr (disc 000) | | Costs 40 yr (disc 000) | |
| --- | --- | --- | --- | --- | --- | --- | --- | --- |
| (baseline values) |  |  |  |  | Projected | Change v. prior | Projected | Change v. prior |
| Baseline | n/a | | Current | 12,509 | 112,040 |  | 75.34 |  |
|  |  |  | 350 | 10,598 | 95,864 | -16,176 | 71.48 | -3.86 |
|  |  |  | 500 | 8,655 | 80,606 | -15,258 | 66.52 | -4.96 |
|  |  |  | All CD4 levels | 7,124 | 69,393 | -11,213 | 61.5 | -5.01 |
| Life expectancy off ART (11 yrs) | 14 yrs | | Current | 9,867 | 86,483 |  | 86.29 |  |
|  |  |  | 350 | 8,401 | 74,179 | -12,304 | 80.9 | -5.39 |
|  |  |  | 500 | 6,914 | 62,851 | -11,328 | 74.61 | -6.29 |
|  |  |  | All CD4 levels | 5,828 | 55,163 | -7,688 | 69.04 | -5.58 |
| Life expectancy increase on ART (x2.5) | X3.5 | | Current | 11,325 | 101,044 |  | 81.72 |  |
|  |  |  | 350 | 9,244 | 83,157 | -17,888 | 77.84 | -3.88 |
|  |  |  | 500 | 7,428 | 68,743 | -14,414 | 72.05 | -5.79 |
|  |  |  | All CD4 levels | 6,193 | 59,617 | -9,126 | 66.6 | -5.45 |
| Cotrim (not provided) | (provided on diagnosis) | | Current | 11,316 | 100,592 |  | 68.16 |  |
|  |  |  | 350 | 9,843 | 88,463 | -12,129 | 64.79 | -3.37 |
|  |  |  | 500 | 8,157 | 75,699 | -12,764 | 60 | -4.79 |
|  |  |  | All CD4 levels | 6,719 | 65,530 | -10,169 | 54.8 | -5.2 |
| Residual transmission on ART (8%) | 1% | | Current | 12,336 | 110,806 |  | 74.48 |  |
|  |  |  | 350 | 10,305 | 93,830 | -16,975 | 69.84 | -4.64 |
|  |  |  | 500 | 8,200 | 77,554 | -16,276 | 63.46 | -6.38 |
|  |  |  | All CD4 levels | 6,620 | 66,125 | -11,429 | 56.92 | -6.53 |
|  | 15% | | Current | 12,672 | 113,214 |  | 78.67 |  |
|  |  |  | 350 | 10,863 | 97,715 | -15,499 | 74.79 | -3.88 |
|  |  |  | 500 | 9,042 | 83,228 | -14,487 | 70.21 | -4.59 |
|  |  |  | All CD4 levels | 7,572 | 72,316 | -10,912 | 66.01 | -4.2 |
|  | 30% | | Current | 12,998 | 115,559 |  | 80.4 |  |
|  |  |  | 350 | 11,362 | 101,226 | -14,333 | 77.76 | -2.64 |
|  |  |  | 500 | 9,728 | 87,922 | -13,304 | 75.04 | -2.72 |
|  |  |  | All CD4 levels | 8,375 | 77,629 | -10,292 | 73.32 | -1.72 |
| Drop out rate on ART (1.5%) | 3% | | Current | 12,834 | 114,705 |  | 74.25 |  |
|  |  |  | 350 | 11,112 | 100,004 | -14,701 | 69.8 | -4.45 |
|  |  |  | 500 | 9,405 | 86,441 | -13,563 | 64.81 | -4.99 |
|  |  |  | All CD4 levels | 7,997 | 76,027 | -10,413 | 61.02 | -3.79 |
|  | 5% | | Current | 13,199 | 117,735 |  | 72.99 |  |
|  |  |  | 350 | 11,683 | 104,659 | -13,076 | 67.76 | -5.22 |
|  |  |  | 500 | 10,228 | 92,933 | -11,726 | 62.42 | -5.35 |
|  |  |  | All CD4 levels | 8,995 | 83,668 | -9,265 | 59.53 | -2.88 |
| First to Second line transition rate on ART (3%) | 1.50% | | Current | 12,509 | 112,040 |  | 74.02 |  |
|  |  |  | 350 | 10,598 | 95,864 | -16,176 | 69.65 | -4.36 |
|  |  |  | 500 | 8,655 | 80,606 | -15,258 | 64.31 | -5.35 |
|  |  |  | All CD4 levels | 7,124 | 69,393 | -11,213 | 59.16 | -5.15 |
|  | 4.50% | | Current | 12,509 | 112,040 |  | 76.48 |  |
|  |  |  | 350 | 10,598 | 95,864 | -16,176 | 73.06 | -3.42 |
|  |  |  | 500 | 8,655 | 80,606 | -15,258 | 68.43 | -4.63 |
|  |  |  | All CD4 levels | 7,124 | 69,393 | -11,213 | 63.53 | -4.9 |
| Inpatient days reduction on ART (7.1 to 1.7) | 9.7 to 1.6 | | Current | 12,509 | 112,040 |  | 84.29 |  |
|  |  |  | 350 | 10,598 | 95,864 | -16,176 | 76.5 | -7.79 |
|  |  |  | 500 | 8,655 | 80,606 | -15,258 | 69.14 | -7.36 |
|  |  |  | All CD4 levels | 7,124 | 69,393 | -11,213 | 63.4 | -5.74 |
|  | 4.5 to 1.8 | | Current | 12,509 | 112,040 |  | 63.75 |  |
|  |  |  | 350 | 10,598 | 95,864 | -16,176 | 63.41 | -0.34 |
|  |  |  | 500 | 8,655 | 80,606 | -15,258 | 61.07 | -2.34 |
|  |  |  | All CD4 levels | 7,124 | 69,393 | -11,213 | 57.26 | -3.81 |
| PHC visits pre and on ART, routine and for OI and with TB (median values) | Low values | | Current | 12,509 | 112,040 |  | 67.25 |  |
|  |  |  | 350 | 10,598 | 95,864 | -16,176 | 63.76 | -3.49 |
|  |  |  | 500 | 8,655 | 80,606 | -15,258 | 59.5 | -4.26 |
|  |  |  | All CD4 levels | 7,124 | 69,393 | -11,213 | 55.42 | -4.08 |
|  | High values | | Current | 12,509 | 112,040 |  | 83.43 |  |
|  |  |  | 350 | 10,598 | 95,864 | -16,176 | 79.2 | -4.23 |
|  |  |  | 500 | 8,655 | 80,606 | -15,258 | 73.53 | -5.66 |
|  |  |  | All CD4 levels | 7,124 | 69,393 | -11,213 | 67.59 | -5.94 |
|  | Low value | |  |  | Projected | Change vs prior | Projected | Change vs prior |
| Cotrim morbidity reduction (0.69) | 0.53 | | Current | 11,316 | 100,592 |  | 70.35 |  |
|  |  |  | 350 | 9,843 | 88,463 | -12,129 | 65.06 | -5.3 |
|  |  |  | 500 | 8,157 | 75,699 | -12,764 | 59.04 | -6.01 |
|  |  |  | All CD4 levels | 6,719 | 65,530 | -10,169 | 53.43 | -5.62 |
|  | 0.84 | | Current | 11,316 | 100,592 |  | 65.97 |  |
|  |  |  | 350 | 9,843 | 88,463 | -12,129 | 64.53 | -1.44 |
|  |  |  | 500 | 8,157 | 75,699 | -12,764 | 60.95 | -3.58 |
|  |  |  | All CD4 levels | 6,719 | 65,530 | -10,169 | 56.17 | -4.78 |
| ART and testing costs | ART and testing costs at SA levels | | Current | 12,509 | 112,040 |  | 96.95 |  |
| (SMCS prices) |  |  | 350 | 10,598 | 95,864 | -16,176 | 100 | 3.05 |
|  |  |  | 500 | 8,655 | 80,606 | -15,258 | 100.13 | 0.14 |
|  |  |  | All CD4 levels | 7,124 | 69,393 | -11,213 | 96.38 | -3.76 |
|  | ART costs at SA levels | | Current | 12,509 | 112,040 |  | 89.15 |  |
|  |  |  | 350 | 10,598 | 95,864 | -16,176 | 90.72 | 1.57 |
|  |  |  | 500 | 8,655 | 80,606 | -15,258 | 89.89 | -0.83 |
|  |  |  | All CD4 levels | 7,124 | 69,393 | -11,213 | 86.13 | -3.77 |
|  | Testing costs at SA levels | | Current | 12,509 | 112,040 |  | 83.14 |  |
|  |  |  | 350 | 10,598 | 95,864 | -16,176 | 80.75 | -2.38 |
|  |  |  | 500 | 8,655 | 80,606 | -15,258 | 76.76 | -4 |
|  |  |  | All CD4 levels | 7,124 | 69,393 | -11,213 | 71.76 | -5 |
| Inpatient day costs for level 1, 2 and 3 hospitals (median values) | Low | | Current | 12,509 | 112,040 |  | 65.71 |  |
|  |  |  | 350 | 10,598 | 95,864 | -16,176 | 63.65 | -2.06 |
|  |  |  | 500 | 8,655 | 80,606 | -15,258 | 60.42 | -3.23 |
|  |  |  | All CD4 levels | 7,124 | 69,393 | -11,213 | 56.61 | -3.81 |
|  | High | | Current | 12,509 | 112,040 |  | 83.97 |  |
|  |  |  | 350 | 10,598 | 95,864 | -16,176 | 78.13 | -5.85 |
|  |  |  | 500 | 8,655 | 80,606 | -15,258 | 71.35 | -6.78 |
|  |  |  | All CD4 levels | 7,124 | 69,393 | -11,213 | 65.32 | -6.03 |
| Unfavourable case: | | | Current | 14,669 | 130,927 |  | 120.79 |  |
| Full SA costs, low testing and coverage, high refusals & regimen change | | | 350 | 13,702 | 122,826 | -8,102 | 117.92 | -2.87 |
|  | | | 500 | 12,709 | 114,913 | -7,912 | 115.95 | -1.97 |
|  | | | All CD4 levels | 11,447 | 105,294 | -9,620 | 116.5 | 0.55 |
| Full SA case: | | | Current | 12,509 | 112,040 |  | 117.86 |  |
| ART, testing and inpatient days as SA 2009 | | | 350 | 10,598 | 95,864 | -16,176 | 114.26 | -3.6 |
|  | | | 500 | 8,655 | 80,606 | -15,258 | 109.74 | -4.53 |
|  | | | All CD4 levels | 7,124 | 69,393 | -11,213 | 104.05 | -5.69 |
| Favourable case: | | | Current | 11,068 | 99,149 |  | 60.67 |  |
| Low ARV and testing costs, high coverage and testing rate, low refusals and resistance | | | 350 | 8,893 | 80,650 | -18,499 | 61.4 | 0.74 |
|  | | | 500 | 6,987 | 65,697 | -14,953 | 58.79 | -2.62 |
|  | | | All CD4 levels | 5,791 | 56,926 | -8,770 | 54.8 | -3.98 |
| Testing rate (annual) | | 2 yrs | Current | 13,594 | 121,299 |  | 75.47 |  |
|  |  |  | 350 | 12,085 | 108,818 | -12,481 | 72.69 | -2.78 |
|  |  |  | 500 | 10,566 | 96,946 | -11,872 | 69.28 | -3.41 |
|  |  |  | All CD4 levels | 8,820 | 83,967 | -12,979 | 65.11 | -4.17 |
| Baseline | | n/a | Current | 12,509 | 112,040 |  | 75.34 |  |
|  |  |  | 350 | 10,598 | 95,864 | -16,176 | 71.48 | -3.86 |
|  |  |  | 500 | 8,655 | 80,606 | -15,258 | 66.52 | -4.96 |
|  |  |  | All CD4 levels | 7,124 | 69,393 | -11,213 | 61.5 | -5.01 |
| Life expectancy off ART (11 yrs) | | 14 yrs | Current | 9,867 | 86,483 |  | 86.29 |  |
|  |  |  | 350 | 8,401 | 74,179 | -12,304 | 80.9 | -5.39 |
|  |  |  | 500 | 6,914 | 62,851 | -11,328 | 74.61 | -6.29 |
|  |  |  | All CD4 levels | 5,828 | 55,163 | -7,688 | 69.04 | -5.58 |
| Life expectancy increase on ART (x2.5) | | X3.5 | Current | 11,325 | 101,044 |  | 81.72 |  |
|  |  |  | 350 | 9,244 | 83,157 | -17,888 | 77.84 | -3.88 |
|  |  |  | 500 | 7,428 | 68,743 | -14,414 | 72.05 | -5.79 |
|  |  |  | All CD4 levels | 6,193 | 59,617 | -9,126 | 66.6 | -5.45 |
| Cotrim (not provided) | | (provided on diagnosis) | Current | 11,316 | 100,592 |  | 68.16 |  |
|  |  |  | 350 | 9,843 | 88,463 | -12,129 | 64.79 | -3.37 |
|  |  |  | 500 | 8,157 | 75,699 | -12,764 | 60 | -4.79 |
|  |  |  | All CD4 levels | 6,719 | 65,530 | -10,169 | 54.8 | -5.2 |
| Residual transmission on ART (8%) | | 1% | Current | 12,336 | 110,806 |  | 74.48 |  |
|  |  |  | 350 | 10,305 | 93,830 | -16,975 | 69.84 | -4.64 |
|  |  |  | 500 | 8,200 | 77,554 | -16,276 | 63.46 | -6.38 |
|  |  |  | All CD4 levels | 6,620 | 66,125 | -11,429 | 56.92 | -6.53 |
|  |  | 15% | Current | 12,672 | 113,214 |  | 78.67 |  |
|  |  |  | 350 | 10,863 | 97,715 | -15,499 | 74.79 | -3.88 |
|  |  |  | 500 | 9,042 | 83,228 | -14,487 | 70.21 | -4.59 |
|  |  |  | All CD4 levels | 7,572 | 72,316 | -10,912 | 66.01 | -4.2 |
|  |  | 30% | Current | 12,998 | 115,559 |  | 80.4 |  |
|  |  |  | 350 | 11,362 | 101,226 | -14,333 | 77.76 | -2.64 |
|  |  |  | 500 | 9,728 | 87,922 | -13,304 | 75.04 | -2.72 |
|  |  |  | All CD4 levels | 8,375 | 77,629 | -10,292 | 73.32 | -1.72 |
| Drop out rate on ART (1.5%) | | 3% | Current | 12,834 | 114,705 |  | 74.25 |  |
|  |  |  | 350 | 11,112 | 100,004 | -14,701 | 69.8 | -4.45 |
|  |  |  | 500 | 9,405 | 86,441 | -13,563 | 64.81 | -4.99 |
|  |  |  | All CD4 levels | 7,997 | 76,027 | -10,413 | 61.02 | -3.79 |
|  |  | 5% | Current | 13,199 | 117,735 |  | 72.99 |  |
|  |  |  | 350 | 11,683 | 104,659 | -13,076 | 67.76 | -5.22 |
|  |  |  | 500 | 10,228 | 92,933 | -11,726 | 62.42 | -5.35 |
|  |  |  | All CD4 levels | 8,995 | 83,668 | -9,265 | 59.53 | -2.88 |
| First to Second line transition rate on ART (3%) | | 1.50% | Current | 12,509 | 112,040 |  | 74.02 |  |
|  |  |  | 350 | 10,598 | 95,864 | -16,176 | 69.65 | -4.36 |
|  |  |  | 500 | 8,655 | 80,606 | -15,258 | 64.31 | -5.35 |
|  |  |  | All CD4 levels | 7,124 | 69,393 | -11,213 | 59.16 | -5.15 |
|  |  | 4.50% | Current | 12,509 | 112,040 |  | 76.48 |  |
|  |  |  | 350 | 10,598 | 95,864 | -16,176 | 73.06 | -3.42 |
|  |  |  | 500 | 8,655 | 80,606 | -15,258 | 68.43 | -4.63 |
|  |  |  | All CD4 levels | 7,124 | 69,393 | -11,213 | 63.53 | -4.9 |
| Inpatient days reduction on ART (7.1 to 1.7) | | 9.7 to 1.6 | Current | 12,509 | 112,040 |  | 84.29 |  |
|  |  |  | 350 | 10,598 | 95,864 | -16,176 | 76.5 | -7.79 |
|  |  |  | 500 | 8,655 | 80,606 | -15,258 | 69.14 | -7.36 |
|  |  |  | All CD4 levels | 7,124 | 69,393 | -11,213 | 63.4 | -5.74 |
|  |  | 4.5 to 1.8 | Current | 12,509 | 112,040 |  | 63.75 |  |
|  |  |  | 350 | 10,598 | 95,864 | -16,176 | 63.41 | -0.34 |
|  |  |  | 500 | 8,655 | 80,606 | -15,258 | 61.07 | -2.34 |
|  |  |  | All CD4 levels | 7,124 | 69,393 | -11,213 | 57.26 | -3.81 |
| PHC visits pre and on ART, routine and for OI and with TB (median values) | | Low values | Current | 12,509 | 112,040 |  | 67.25 |  |
|  |  |  | 350 | 10,598 | 95,864 | -16,176 | 63.76 | -3.49 |
|  |  |  | 500 | 8,655 | 80,606 | -15,258 | 59.5 | -4.26 |
|  |  |  | All CD4 levels | 7,124 | 69,393 | -11,213 | 55.42 | -4.08 |
|  |  | High values | Current | 12,509 | 112,040 |  | 83.43 |  |
|  |  |  | 350 | 10,598 | 95,864 | -16,176 | 79.2 | -4.23 |
|  |  |  | 500 | 8,655 | 80,606 | -15,258 | 73.53 | -5.66 |
|  |  |  | All CD4 levels | 7,124 | 69,393 | -11,213 | 67.59 | -5.94 |
|  |  | Low value |  |  | Projected | Change vs prior | Projected | Change vs prior |
| Cotrim morbidity reduction (0.69) | | 0.53 | Current | 11,316 | 100,592 |  | 70.35 |  |
|  |  |  | 350 | 9,843 | 88,463 | -12,129 | 65.06 | -5.3 |
|  |  |  | 500 | 8,157 | 75,699 | -12,764 | 59.04 | -6.01 |
|  |  |  | All CD4 levels | 6,719 | 65,530 | -10,169 | 53.43 | -5.62 |
|  |  | 0.84 | Current | 11,316 | 100,592 |  | 65.97 |  |
|  |  |  | 350 | 9,843 | 88,463 | -12,129 | 64.53 | -1.44 |
|  |  |  | 500 | 8,157 | 75,699 | -12,764 | 60.95 | -3.58 |
|  |  |  | All CD4 levels | 6,719 | 65,530 | -10,169 | 56.17 | -4.78 |
| ART and testing costs | | ART and testing costs at SA levels | Current | 12,509 | 112,040 |  | 96.95 |  |
| (SMCS prices) | |  | 350 | 10,598 | 95,864 | -16,176 | 100 | 3.05 |
|  | |  | 500 | 8,655 | 80,606 | -15,258 | 100.13 | 0.14 |
|  | |  | All CD4 levels | 7,124 | 69,393 | -11,213 | 96.38 | -3.76 |
|  | | ART costs at SA levels | Current | 12,509 | 112,040 |  | 89.15 |  |
|  | |  | 350 | 10,598 | 95,864 | -16,176 | 90.72 | 1.57 |
|  | |  | 500 | 8,655 | 80,606 | -15,258 | 89.89 | -0.83 |
|  | |  | All CD4 levels | 7,124 | 69,393 | -11,213 | 86.13 | -3.77 |
|  | | Testing costs at SA levels | Current | 12,509 | 112,040 |  | 83.14 |  |
|  | |  | 350 | 10,598 | 95,864 | -16,176 | 80.75 | -2.38 |
|  | |  | 500 | 8,655 | 80,606 | -15,258 | 76.76 | -4 |
|  | |  | All CD4 levels | 7,124 | 69,393 | -11,213 | 71.76 | -5 |
| Inpatient day costs for level 1, 2 and 3 hospitals (median values) | | Low | Current | 12,509 | 112,040 |  | 65.71 |  |
|  |  |  | 350 | 10,598 | 95,864 | -16,176 | 63.65 | -2.06 |
|  |  |  | 500 | 8,655 | 80,606 | -15,258 | 60.42 | -3.23 |
|  |  |  | All CD4 levels | 7,124 | 69,393 | -11,213 | 56.61 | -3.81 |
|  |  | High | Current | 12,509 | 112,040 |  | 83.97 |  |
|  |  |  | 350 | 10,598 | 95,864 | -16,176 | 78.13 | -5.85 |
|  |  |  | 500 | 8,655 | 80,606 | -15,258 | 71.35 | -6.78 |
|  |  |  | All CD4 levels | 7,124 | 69,393 | -11,213 | 65.32 | -6.03 |
| Unfavourable case: | | | Current | 14,669 | 130,927 |  | 120.79 |  |
| Full SA costs, low testing and coverage, high refusals & regimen change | | | 350 | 13,702 | 122,826 | -8,102 | 117.92 | -2.87 |
|  | | | 500 | 12,709 | 114,913 | -7,912 | 115.95 | -1.97 |
|  | | | All CD4 levels | 11,447 | 105,294 | -9,620 | 116.5 | 0.55 |
| Full SA case: | | | Current | 12,509 | 112,040 |  | 117.86 |  |
| ART, testing and inpatient days as SA 2009 | | | 350 | 10,598 | 95,864 | -16,176 | 114.26 | -3.6 |
|  | | | 500 | 8,655 | 80,606 | -15,258 | 109.74 | -4.53 |
|  | | | All CD4 levels | 7,124 | 69,393 | -11,213 | 104.05 | -5.69 |
| Favourable case: | | | Current | 11,068 | 99,149 |  | 60.67 |  |
| Low ARV and testing costs, high coverage and testing rate, low refusals and resistance | | | 350 | 8,893 | 80,650 | -18,499 | 61.4 | 0.74 |
|  | | | 500 | 6,987 | 65,697 | -14,953 | 58.79 | -2.62 |
|  | | | All CD4 levels | 5,791 | 56,926 | -8,770 | 54.8 | -3.98 |
| Testing rate (annual) | | 2 yrs | Current | 13,594 | 121,299 |  | 75.47 |  |
|  |  |  | 350 | 12,085 | 108,818 | -12,481 | 72.69 | -2.78 |
|  |  |  | 500 | 10,566 | 96,946 | -11,872 | 69.28 | -3.41 |
|  |  |  | All CD4 levels | 8,820 | 83,967 | -12,979 | 65.11 | -4.17 |
|  |  |  |  |  |  |  |  |  |
|  |  | 3 yrs | Current | 14,139 | 125,978 |  | 76.34 |  |
|  |  |  | 350 | 13,090 | 117,389 | -8,589 | 73.54 | -2.8 |
|  |  |  | 500 | 12,052 | 109,344 | -8,045 | 71.59 | -1.95 |
|  |  |  | All CD4 levels | 10,618 | 98,731 | -10,613 | 69.23 | -2.36 |
| Testing coverage (90%) | | 95% | Current | 12,478 | 111,788 |  | 78.35 |  |
|  |  |  | 350 | 10,561 | 95,546 | -16,241 | 73.65 | -4.69 |
|  |  |  | 500 | 8,609 | 80,217 | -15,329 | 67.81 | -5.85 |
|  |  |  | All CD4 levels | 7,092 | 69,107 | -11,110 | 62.18 | -5.63 |
|  |  | 80% | Current | 12,572 | 112,558 |  | 76.65 |  |
|  |  |  | 350 | 10,679 | 96,570 | -15,988 | 72.32 | -4.33 |
|  |  |  | 500 | 8,759 | 81,497 | -15,073 | 66.84 | -5.48 |
|  |  |  | All CD4 levels | 7,200 | 70,060 | -11,437 | 61.37 | -5.48 |

# 6.0 The role of the acute phase

The extent to which ART (anti-retroviral therapy) will reduce transmission of HIV has received considerable attention in recent years since the publication of two modelling studies that suggests that ART could be used to effectively stop the epidemic of HIV. Using data from studies of the relationship between transmission and viral load we show that transmission saturates at high viral loads. We fit the data to two models, a widely used model based on a power-law and a new model based on an exponential converging to an asymptote. We show that the ART is likely to reduce transmission by 91.6% (81.7%−96.2%) under the first model and 99.5% (98.5%−99.8%) under the second model.

There has also been considerable debate concerning the role of the acute phase in the transmission of HIV. High levels of transmission during the acute phase have been used to argue that this will significantly compromise the impact of treatment on preventing new infections[^16^](#_ENREF_16) and has also been used to argue that high levels of concurrency during the acute phase are an important driver of the epidemic.[^17^](#_ENREF_17) We show that increased viral load during the acute phase is unlikely to contribute significantly to the spread of HIV. We argue that a much more important feature of HIV-epidemiology is the variability in the infectiousness of HIV-positive people and the variability in the susceptibility of HIV-negative people, neither of which have been adequately addressed in population models of HIV-transmission. Finally, we show that even if a significant proportion of infections were transmitted during the acute phase, this would not significantly compromise the impact of treatment on population levels of transmission given the constraint implied by the doubling time of the epidemic.

Understanding the relationship between HIV viral load and transmission is essential if we are to understand the dynamics of HIV and, in particular, the impact of anti-retroviral treatment (ART) on HIV transmission. It is generally accepted that the risk of HIV-transmission increases as the plasma viral load increases. However, data that can be used to address this question are few, especially at very high and very low viral loads. Transmission at low viral loads will determine the impact that anti-retroviral therapy has on transmission; transmission at high viral loads will determine the importance of transmission during the acute phase of HIV infection; transmission at intermediate viral loads will determine the rate at which HIV spreads through a population.

The data show clearly that transmission increases more slowly than viral load and most attempts to establish a relationship between transmission and viral load assume a power-law relationship. Here we present an alternative model in which we assume that transmission increases linearly with viral load when the viral load is low but converges to an asymptote at high viral loads. While our model is easier to justify biologically, an explanation is needed for the saturation effect in both models. Either better data or a better understanding of the mechanism of infection are needed to distinguish between the two models; the available data are not sufficient to distinguish between the two models on statistical grounds.

We then examine data on the dynamics of viral load during the acute phase and the chronic phase in order to establish the duration and magnitude of the viral load during the acute phase of infection. This enables us to estimate the proportion of transmission events that take place during the acute phase of infection and we attempt to reconcile our estimates with previous estimates and claims.

We are then able to estimate the likely impact of ART on HIV transmission. Finally, we consider the consequences that would follow if transmission during the acute phase was as high as has been estimated on some studies in order to determine the impact that treatment would have on transmission under these conditions.

## 6.1 Data sources

Attempts to measure the relationship between viral load and transmission have either presented data as the risk per sexual contact or as the risk per unit time and have included studies of discordant couples[^19-26^](#_ENREF_19) and vertical transmission from mothers to their children.[^27^](#_ENREF_27) Here we consider three studies reporting data on the risk of transmission per unit time: a meta-analysis of studies published before 2009;[^22^](#_ENREF_22) a study of the impact of treating HSV-2 on HIV-1 transmission which included measurements of viral load;[^28^](#_ENREF_28) data on sero-discordant couples in East and Southern Africa.[^29^](#_ENREF_29)

We consider data from several different sources. For the frequency distribution of viral load among people in a severe HIV epidemic we used measurements of viral load measured in a cross-sectional survey of young men in Orange Farm, South Africa.[^30^](#_ENREF_30)

To determine the relationship between transmission and plasma viral load we use data from three studies. The first by Attia *et al.*[^22^](#_ENREF_22) is a meta-analysis of 6 earlier studies, the second by Donnell *et al*.[^28^](#_ENREF_28) is a study of the impact of treating HSV-2 on HIV-1 transmission, the third by Lingappa *et al.*[^29^](#_ENREF_29) was based on prospective data collected from 2004 to 2008 in East and Southern Africa among HIV-1 sero-discordant couples. To determine the relationship between transmission and viral load in genital secretions we use data from a prospective study in Africa of 2521 heterosexual sero-discordant couples in Africa where Baeten *et al.*[^31^](#_ENREF_31) considered male to female and female to male transmission separately. It is important to note that the data are all measured as the number of transmission events per year which depends, in turn, on the frequency of sexual encounters.

## 6.2 Methods

### 6.2.1 Estimating the distribution of viral load

A problem all of the studies reporting the relationship between transmission and viral load is that the authors only publish the number of transmission events ion different ranges of viral load and we need to estimate the mean viral load for each range. To do this we start from the data in Figure 2 and determine the Box-Cox transformation[^32^](#_ENREF_32)

,

where *w* is the transformed values of the viral load *v*, that gives the best fit to the data assuming that the transformed data is normally distributed. The Box-Cox parameter, *λ*, is 2.58. The resulting distribution can be fitted to a normal distribution with a mean value of 20.8 and a standard deviation of 9.4. As described in 7.0 Appendix 1 below we then assume that in the study by Attia *et al.*[^22^](#_ENREF_22) the density function is the same as in Orange Farm while in the study by Donnell *et al*.[^28^](#_ENREF_28) we keep the same value of the Box-Cox parameter but fit a cumulative normal distribution to the observed numbers of people in each range of the viral load. For the Orange Farm study the mode of the viral load distribution on a log-scale is at 95k virions/mL while in the Donnell *et al*.[^28^](#_ENREF_28) study 2 it is appears to be rather lower at 29k virions/mL.

### 6.2.2 Models for transmission as a function of viral load

We use two models to fit the data on transmission as a function of viral load. One model, which is used in all four of the publications cited, assumes a power-law relationship between transmission and viral load; the second model, which we develop here, assumes that at very low viral loads transmission increases linearly with viral load and converges exponentially to an asymptote when the viral load is very high. The equation for the power-law model is

$$T=\beta V^{\sigma}$$

and for the converging-exponential model is

$T=\alpha\left(1-\mathrm{e}^{\rho V}\right)$.

At low values of the viral load Equation reduces to

$$T\approx\alpha\rho V$$

Figure 2. Frequency distribution of viral loads in young men from a cross-sectional survey carried out in Orange Farm, South Africa.[^30^](#_ENREF_30)

## 6.3 Results

### 6.3.1 Distribution of viral load

The distribution of viral load before people start ART is variable but seldom published. The data in Figure 2, measured in a cross-sectional survey of young men in Orange Farm, South Africa,[^30^](#_ENREF_30) shows that the mode of the distribution is at 53k/mL but with 95% of the distribution lying between 224/mL and 882k/mL, spanning more than three orders of magnitude. Those with a viral load in the region of 10^6^/mL will be more infectious than those with a viral load of less then 100/μL. But not only will treating those with the highest viral load have the greatest individual benefit, since survival decreases as viral load increases, but it will also have the greatest public health benefit by reducing transmission.

Figure 3. Two models fitted to data on the annual risk of infection in a discordant couple as a function of plasma viral load. Left: Converging-exponential model (Equation ). Right: Power law model (Equation ). Data A and B: Attia *et al.*[^22^](#_ENREF_22); C and D: Donnell *et al.*[^33^](#_ENREF_33); E and F Lingappa *et al.*[^29^](#_ENREF_29) The fitted parameters and the significance levels for the fits are A: *α *= 0.083 (0.062−0.107) infections per year, *ρ* = 2.23 (2.14−2.31) mL/virion, *p* = 0.80; B: *β* = 0.32 (0.30−0.33), *σ* = 0.0019 (00.15−0.023), 0.0006; C: *α* = 0.032 (0.024−0.042) infections per year, *ρ* = 2.31 (2.25−2.37) mL/virion, *p* = 0.38; D: *β* = 0.45 (0.42−0.47), *σ* = 0.00017 (0.00013−0.00022), 0.30; E: *α* = 0.053 (0.042−0.066) infections per year, *ρ* = 2.35 (2.30−2.39) mL/virion, *p* = 0.11; F: *β* = 0.43 (0.41−0.45), *σ* = 0.00027 (0.00021−0.00034), *p* = 0.39.

## 6.3.2 Transmission as a function of plasma viral load

Figure 3 shows the data and the fitted curves for the three studies of transmission as a function of plasma viral load fitted to the two models. For the data from Attia *et al.*[^22^](#_ENREF_22) (Figure 3 A and B) the exponential model give a statistically good fit (*p* = 0.8) while the power-law model does not (*p* = 0.0008). For the data from Donnell *et al*.[^28^](#_ENREF_28) (Figure 3 C and D) and Lingappa *et al.*[^29^](#_ENREF_29) (Figure 3 E and F) both models give statistically acceptable fits. On the basis of these data alone one cannot say with confidence that either model is better.

Table 5. Model 1 assumes that transmission increases linearly with viral load at low viral loads but converges exponentially to an asymptote. Model II assumes a power law relationship between transmission and viral load. *RR* gives the risk of transmission at a viral load of 100/μL compared to that at 10^5^/mL. SVL is the saturation viral load (see text for details). Power is the power in the power law relation between transmission and viral load. Data plotted in Figure 3. The sets of data in each of the first three rows are not significantly different (*p* > 0.67 in all cases) but the data in the bottom row are significantly over-dispersed (*p* < 0.0001). Mean values are therefore weighted means using the estimated errors for the first three rows and unweighted means with the error calculated from the residuals in the bottom row.

| Model | | Attia *et al.*[^22^](#_ENREF_22) | Donnell *et al.*[^33^](#_ENREF_33) | Lingappa *et al.*[^29^](#_ENREF_29) | Mean |
| --- | --- | --- | --- | --- | --- |
| I | *RR* (%) | 0.91 (0.38−2.43) | 0.46 (0.20−1.37) | 0.37 (0.18−1.01) | 0.53 (0.18−1.52) |
|  | Log_10_(SVL) | 4.04 (3.69−4.36) | 4.39 (4.12−4.65) | 4.56 (4.33−4.76) | 4.42 (4.40−4.44) |
| II | *RR* (%) | 14.05 (7.76−31.4) | 6.19 (3.08−13.0) | 6.87 (3.60−12.80) | 8.35 (3.80−18.3) |
|  | Power | 0.32 (0.30−0.33) | 0.45 (0.42−0.47) | 0.43 (0.41−0.45) | 0.32 (0.30−0.34) |

The key features of the fits in Figure 3 are summarized in Table 5. To obtain an estimate of the reduction in transmission as viral load falls we compare the risk of infection at a viral load of 100/μL, which should be achievable under anti-retroviral therapy, with the risk at a viral load of 50k/mL, corresponding to the mode of the distribution.

Under the converging-exponential model reducing the viral load from 50k/mL to 100/ml gives a risk ratio of 0.53% (0.18% to 1.52%) reducing transmission by 189 (66 to 556) times. Using the power-law model the corresponding risk ratio is 8.4% (3.8% to 18.3%) reducing transmission by 12 (5 to 26) times. Both give substantial reductions in transmission but it will be important to establish which one is better since the extrapolation to low values of viral load depends critically on the choice of model.

Under the converging-exponential model, transmission saturates when the log-viral load is greater than 4.42 (4.40−4.44) for which the viral load is 26.0 (24.8−27.3) k/mL. Under the power-law model, transmission increases as the viral load to the power of 0.32 (0.30−0.34), so that the increase is less than linear. In either case a biological explanation for the fact that transmission saturates at high values of viral load needs to be explained.

## 6.3.3 Transmission as a function of genital viral load

The viral load in genital secretions is likely to be more important than the viral load in plasma in determining transmission. Figure 4 shows some limited data and the fitted curves for the relationship between transmission and viral load in genital secretions. The data fit both models acceptably well so that again one cannot determine which model is better on the basis of the statistical fit.

102103104105106107102103104105106107

1.0000

0.1000

0.0100

0.0010

0.0001

1.0000

0.1000

0.0100

0.0010

0.0001

Viral load (copies/mL) Viral load (copies/mL)

Infections/yr Infections/yr

1.0000

0.1000

0.0100

0.0010

0.0001

1.0000

0.1000

0.0100

0.0010

0.0001

102103104105106107102103104105106107

A B

C D

Figure 4. Two models fitted to data on the annual risk of infection in a discordant couple as a function of viral load in genital secretions.[^31^](#_ENREF_31) Left: Converging-exponential model (Equation ). Right: Power law model (Equation ). Data A and B: female to male transmission:; C and D: male to female transmission. A: *α *= 0.025 (0.017−0.035) infections per year, *ρ* = 1.55 (1.28−1.78) mL/virion, *p* = 0.16; B: *β* = 0.23 (0.21−0.24), *σ* = 0.0030 (00.26−0.035), 0.96; C: *α* = 0.047 (0.030−0.071) infections per year, *ρ* = 1.62 (1.36−1.82) mL/virion, *p* = 0.55; D: *β* = 0.21 (0.15−0.25), *σ* = 0.0067 (0.0042−0.0101), *p* = 0.22.

The key features of the fits in Figure 4 are summarized in Table 6. The asymptotes are slightly but not significantly lower than the asymptotes in Figure 3 but the data in Figure 4 suggest that the saturation viral load in genital secretions is 197 (90−430) times, or about two orders of magnitude, less than in the plasma. Using the power-law model gives a correspondingly low value for the power in the fit. The reasons for the saturation effect remain to be explained. These data are much less precise than the data based on plasma viral load and reducing the genital viral load to 100/μL would reduce transmission by 92.6% (31.9%−99.2%) using Model I and by 84.7% (21%−97.0%) using Model II.

Table 6. Relative risk of transmission at high and low viral loads, the saturation viral load and the power-law relationship between viral load and transmission. Model 1 assumes that transmission increases linearly with viral load at low viral loads but converges exponentially to an asymptote. Model II assumes a power law relationship between transmission and viral load. *RR* gives the risk of transmission at a viral load of 10/μL compared to that at 10^5^/mL. SVL is the saturation viral load (see text for details). Power is the power in the power law relation between transmission and viral load. The estimates do not differ significantly; *p* > 0.95 in all cases.

| Model |  | Baeten *et al.* F to M[^31^](#_ENREF_31) | Baeten *et al.* M to F[^31^](#_ENREF_31) | Mean |
| --- | --- | --- | --- | --- |
| I | *RR* (%) | 8.72 (1.82−37.1) | 6.17 (1.24−32.4) | 7.44 (0.81−68.1) |
|  | Log_10_(SVL) | 2.04 (1.57−2.57) | 2.20 (1.68−2.69) | 2.12 (1.51−2.98) |
| II | *RR* (%) | 14.31 (5.11−40.8) | 17.0 (5.07−74.9) | 15.25 (2.95−79.0) |
|  | Power | 0.23 (0.21−0.24) | 0.21 (0.15−0.25) | 0.23 (0.20−0.26) |

## 6.3.4 Viral load during the acute phase

After a person has been infected with HIV the viral load increases rapidly with a doubling time of 20.5 (18.2–23.4) hours.[^34^](#_ENREF_34) Assuming that each infection is established from a single virion, it will take about 3.5 weeks to reach a concentration of 10^5^ virions per ml. in a person with 5 litres of blood. In order to fully characterize the acute phase, HIV negative people would have to be tested once or twice a week to ensure that the initial rise is accurately captured. An early study, based on data from newly infected plasma donors[^34^](#_ENREF_34) is shown in Figure 5. Using the full-width at half-maximum above the set point (FWHM), on a log-scale, as the measure of duration of the acute phase gives a value of between 12 and 25 days, the peak viraemia is 105.4 = 251k per mm3 and the set point viraemia of 104.1 = 13k per mm^3^.

Figure 5. Viral load as a function of time[^34^](#_ENREF_34) fitted to an exponential converging to an asymptote that determines the peak viraemia followed by a logistic decline to the set point. The two curves represent the longest (25 days) and shortest (12 days) durations of the acute phase that are consistent with the data.

Preliminary, and only partial, results from a particularly important study have been reported by Robb.[^35^](#_ENREF_35) In this study people from high risk populations in Uganda, Kenya, Tanzania and Thailand are tested for HIV twice a week and as soon as they are found to be HIV positive they are followed up intensively for the first month and then for up to five years. A subset of the date, taken from a presentation,[^35^](#_ENREF_35) are shown in Figure 6. Averaged over all of the data sets the FWHM is 17 ± 3 days, the peak viraemia is 106.6±0.4 = 3.0M (1.6M−10.0M) per mm3 and the set point viraemia of 104.5±0.5 = 32k (10k−100k) per mm^3^.

Figure 6. Data from the acute phase study of Robb.[^35^](#_ENREF_35) The data have been extracted from the presentation. The fitted curves are an exponential increasing to an asymptote followed by a logistic decline to a steady state.

In another study Powers *et al.*[^16^](#_ENREF_16) cite data from Pilcher *et al.*[^36^](#_ENREF_36) for which the FWHM is 2.3 weeks and which Powers *et al.*[^16^](#_ENREF_16) interpret as giving an acute phase duration of two weeks (Powers *et al.*,[^16^](#_ENREF_16) supporting information, Figure 1).

Taken together these data suggest that the acute phase lasts for about 2 to 3 weeks, that the log-peak viraemia is 6.0 ± 1.0 and the log-set point viraemia is about 30 times lower at 4.5 ± 0.5.

## 6.3.5 Transmission during the acute phase

It has been suggested that transmission in the acute phase contributes a significant proportion of overall transmission. Given the difficulty of finding people during the short acute phase this has important consequences for the impact of treatment on transmission.

We first consider the risk of infection during the acute as compared to the chronic phase of infection. Using the converging-exponential model (Figure 3 A, C and E) and assuming that the set point viral load is 6.0 in the acute phase and 4.5 in the chronic phase, the risk of infection in the acute phase is between 1% and 2% higher than in the chronic phase, and less than 7% higher with 95% certainty, for all three sets of data. Using the power-law model, with a power of 2.3 ( The key features of the fits in Figure 4 are summarized in Table 6. The asymptotes are slightly but not significantly lower than the asymptotes in Figure 3 but the data in Figure 4 suggest that the saturation viral load in genital secretions is 197 (90−430) times, or about two orders of magnitude, less than in the plasma. Using the power-law model gives a correspondingly low value for the power in the fit. The reasons for the saturation effect remain to be explained. These data are much less precise than the data based on plasma viral load and reducing the genital viral load to 100/μL would reduce transmission by 92.6% (31.9%−99.2%) using Model I and by 84.7% (21%−97.0%) using Model II.) suggests that transmission in the acute phase is 2.2 (2.0−2.5) times higher than in the chronic phase. Given that the chronic phase lasts for an average of about 11 years, these data suggest that the proportion of transmission that takes place during the acute phase is between about 0.4%, using the converging-exponential model, and 0.8%, using the power-law model. In either case it is unlikely that the high viral load during the acute phase makes a significant difference to the overall rate of infection.

## 6.3.6 Transmission among discordant couples

It important to reconcile the observation that the relatively high viral load in the acute phase does not contribute significantly to overall transmission with claims, based largely on the data from Rakai in which transmission among discordant couples is 7.2 (3−17) times higher in the first five months than in the chronic stage[^38^](#_ENREF_38) and in other analyses of the same data 26 (13−54) times higher in the first 2.9 months than in the chronic stage[^39^](#_ENREF_39) or 13 times higher in the first 2.5 months after infection than in the chronic stage.[^40^](#_ENREF_40) These estimates are not inconsistent, given the uncertainty in the original data and the different assumptions concerning the duration over which the infectivity is measured. However, if the acute phase lasts for only two weeks the infectivity during this short time would have to be of the order of 70 (30−170) times higher than during the chronic phase.

There are three factors that might help to reconcile these observations. The first is that as Wawer *et al.*[^38^](#_ENREF_38) note, genital ulcer disease is significantly associated with the risk of HIV infection and others have shown that the risk of HIV infection may increased by 6.0 (2.6−14.0) times in those recently infected with *Herpes simplex* (HSV) 1 as compared to those with an established HSV-1 infection or without HSV-1 infection. Given the very high incidence of HSV-1 in parts of Africa[^41^](#_ENREF_41) it is not unlikely that people who are infected from outside the relationship will be infected at about the same time with both HIV-2 and HSV-1 with a corresponding increase in the likelihood that they will infect their sexual partners.

A second factor is that different people are more or less susceptible to HIV-infection.[^42^](#_ENREF_42) Furthermore, there is evidence that young women are at especially high risk of HIV infection for both biological and social reasons and Wawer *et al.*[^38^](#_ENREF_38) show that the risk of infection is 2.2 (1.2−4.0) times higher in those younger then 30 years of age as compared to those older than 30 years of age. It must be the case that in a cohort study those that are most susceptible to infection will be infected first leaving those that are at lower risk of infection to be infected later.

The third factor is the inherent bias in cohort studies of discordant couples. It is clear as shown in Figure 2 and Figure 6 that the set-point viral load varies greatly among people infected with HIV. Since those with high set-point viral loads will be more likely to infect their partners first the transmission probability is expected to fall over time. To obtain an approximate estimate of the importance of this effect we start from the data shown in Figure 2, use the risk of transmission as a function of viral load given in Figure 3A, and let survival in years, *S*, vary as the plasma viral load/mm^3^ according to[^43^](#_ENREF_43)

$S=614V^{-0.42}$ n

Given the viral load distribution in prevalent HIV cases (Figure 2) we can estimate the distribution in incident cases from the survival time as a function of viral load. We can then estimate the probability that the index case will remain alive and the susceptible partner will remain uninfected, as a function of time, and hence the transmission rate as a function of time since the index case was infected. We also need to estimate the initial rate of transmission immediately after the index case is infected. To do this we note that the initial epidemic doubling time in heterosexual epidemics is typically about 15 months[^44^](#_ENREF_44) so that the transmission rate, at the start of the epidemic, is about 0.8 per year. We note in passing that this initial transmission rate implies that after five months 28% of people in discordant relationships should be infected which is not significantly different from the estimate given by Wawer *et al.*[^38^](#_ENREF_38) who found that 10/23 or 43% (23%−66%) of negative partners on discordant couples were infected in the first five months.

Proceeding in this way we are able to obtain the risk of transmission in a discordant couple allowing for the fact that the positive partners with high viral load will both be more likely to infect their partners sooner and will be more likely to die sooner.

Figure 7. The risk of transmission in a discordant couple compared to the risk immediately after the index partner becomes infected as a function of time allowing for the fact that those with high viral load are most infectious but will also die soonest.

Figure 7 shows that after two to three years it is possible that the transmission rates in discordant couples may have fall by a factor of about ten, in agreement with the observation of Wawer *et al.*[^38^](#_ENREF_38) without appeal to high rates of transmission during the acute phase. The results in Figure 7 are meant to show that the variability in the set-point viral load can be used to explain the data reported by Wawer *et al.*[^38^](#_ENREF_38) and are not meant to present precise estimates of the effect.

The conclusion, however, is that in models of transmission it is not necessary to include high rates of infection during the acute phase but should include variability in both the infectiousness of infected partners and the susceptibility of uninfected partners.

# 6.4 The impact of the acute phase on treatment as prevention

The analysis presented in this paper suggests that the duration of the acute phase is too short and the increased risk of infection during the acute phase too small to have a significant impact on the overall dynamics of the epidemic. However, there are those that maintain that the duration of the acute phase may be several months and that during this time the risk of infection per sex act maybe increased by up to about 30 times. Given that this could be the case it behooves us to consider the consequences that this would have for treatment as prevention.

The key point is this: one of the few directly observed parameters concerning the epidemiology of HIV is the initial doubling time which, in South Africa, is 1.25 ± 0.25.[^44^](#_ENREF_44) Since the acute phase lasts for considerably less time than the chronic phase, the greater the relative risk of transmission in the acute phase the smaller must be the value of *R*_0_ to maintain the same initial doubling time. Indeed, if we know the initial doubling time (the growth rate *r* in Equations and in Appendix 3) and we know the relative risk of infection in the acute phase and each of the four chronic phases (*βi*/*β*0 in Equations to in Appendix 3) and the duration of each of the four stages (1/*ρi* in Equations to and ) in Appendix 3, then we can determine the values of the each of the individual *ρi* and hence the value of *R*_0_ (Equation in Appendix 3).

Values of *R*_0_, as a function of the relative risk of transmission during the acute phase and the duration of the acute phase in months, are given in Figure 8A. Without ART the value of *R*_0_ is 5.8 (brown rectangle). With *RR* = 2.1 and *D*AP = 2 mo. (green ellipse) *R*_0_ falls to 5.4. With *RR* = 8.3 and *D*AP = 6 mo. (red ellipse) *R*_0_ falls to 3.0. With *RR* = 26 and *D*AP = 6 mo. (blue ellipse) *R*_0_ falls to 2.3. As expected the higher the rate of transmission during the acute phase the lower the value of *R*_0_.

The boundaries of the ellipses indicate the uncertainty in the point estimates which are considerable. Assuming, as noted above, that in the Hollingsworth *et al.*[^39^](#_ENREF_39) study the high values of *D*AP correspond to low values *RR*, and *vice versa*, we slant the corresponding confidence ellipse at an appropriate angle. This also serves to show that if we let *D*AP = 6 mo.

The Hollingsworth *et al.*[^39^](#_ENREF_39) and the Wawer *et al.*[^38^](#_ENREF_38) estimates are not significantly different.

It is important to note that testing people regularly at intervals of one year, say, is more efficient than testing people randomly but once a year on average. In the latter case some people are never tested and some people are tested more often than is strictly necessary. This is discussed further in 7.1 Appendix 2.

Figure 8. The value of *R*_0_ for an epidemic with a doubling time of 15 months as a function of the duration of the acute phase and the relative transmission rate in the acute phase compared to the chronic phase. Colours correspond to different values of *R*_0_ and the numbers in circles give the values on different contour lines. A without ART; B and C with random testing once a year or twice a year, on average; D and E with regular testing once a year or twice a year. Brown rectangle: *RR* = 1; green ellipse: *RR* = 2.1, *D*AP = 1 mo.; ellipse *RR* = 8.3, *D*AP = 6 mo.; blue ellipse: *RR* = 26, *D*AP= 3 mo. The size of the ellipse indicates the uncertainty in the estimate.

Figure 8B shows what happens if people are tested randomly but once a year on average. With *RR* = 1, the value of *R*_0_ falls to 0.58 (brown rectangle). With *RR* = 2.1 and *D*AP = 2 mo. *R*_0_ falls to 0.61. With *RR* = 8.3 and *D*AP = 6 mo. *R*_0_ falls to 0.84 and with *RR* = 26 and *D*AP = 3 mo. *R*_0_ falls to 0.90. In all three cases *R*_0_ still falls below 1 although with the two higher estimates of *RR* it is only 10% to 20% below 1 which allows little margin of error.

Figure 8C shows what happens if the average testing interval is reduced to six months. With *RR* = 1, *R*_0_ falls to 0.29 (brown rectangle). With *RR* = 2.1 and *D*AP = 2 mo. *R*_0_ falls to 0.34. With *RR* = 8.3 and *D*AP = 6 mo. *R*_0_ falls to 0.61 and with *RR* = 26 and *D*AP = 3 mo. *R*_0_ falls to 0.75. Even in the worst case (*RR* = 26, *D*AP = 3 mo.) *R*_0_ is significantly less than 1.

Figure 8D shows what happens if people are tested regularly once a year. With *RR* = 1, the value of *R*_0_ again falls to 0.29 (brown rectangle). With *RR* = 2.1 and *D*AP = 2 mo. *R*_0_ falls to 0.38. With *RR* = 8.3 and *D*AP = 6 mo. *R*_0_ falls to 0.70 and with *RR* = 26 and *D*AP = 3 mo. *R*_0_ falls to 0.82. Again, even in the worst case (*RR* = 26, *D*AP = 3 mo.) *R*_0_ is significantly less than 1.

Figure 8E shows what happens if people are tested regularly twice a year. With *RR* = 1, *R*_0_ falls to 0.14 (brown rectangle). With *RR* = 2.1 and *D*AP = 2 mo. *R*_0_ falls to 0.21. With *RR* = 8.3 and *D*AP = 6 mo. *R*_0_ falls to 0.45 and with *RR* = 26 and *D*AP = 3 mo. *R*_0_ falls to 0.68. Even in the worst case (*RR* = 26, *D*AP  = 3 mo.) *R*_0_ is again significantly less than 1.

We have three estimates of *RR*, the relative risk of infection, and *D*AP, the duration of the acute phase ranging from 2.1 over 2 months to 26.2 over 3 months giving values of *R*_0_ ranging from 5.8 to 2.3. The high estimates for *RR* may well be over-estimates. They are both based on the data from Rakai[^38^](#_ENREF_38) and discordant couple studies in which one partner is ‘sero-prevalent’ will select against those couples who are most infectious and therefore no longer sero-discordant. Furthermore, the high values of *RR* with long values of *D*AP imply values of *R*_0_ ≈ 2 which seems unlikely; if this were the case HIV should be relatively easy to eliminate through minor changes in behaviour and the epidemic should be much less stable.

However, it is clear from this analysis that even if we adopt the most pessimistic view and assume the relative risk of infection is 26 times higher during an acute phase that lasts for 3 months annual testing and immediate treatment has the potential to *R*_0_ to less than 1 and with any further contribution to prevention will probably guarantee elimination in the long term. Testing people regularly, on an annual basis, is considerably more effective than random testing because under random testing some people will be tested very frequently, which is not necessary, while others will be tested very infrequently which is not ideal. With regular testing even the most pessimistic view reduces *R*_0_ to 0.82 and will probably lead to elimination. As expected, testing people twice a year reduces *R*_0_ even further and under all assumptions about the acute phase would guarantee elimination.

However, this analysis raises a possibility that may be even more important than considerations of high rates of infection during the acute phase. We know that the set point values of the viral load vary by several orders of magnitude. In the Fiebig[^34^](#_ENREF_34) study the log_10_(viral load) varies from about 2.6 to 5.6 in the chronic stage.

The data in Figure 8 suggest that variation in the average viral load in a cohort of people, as a function of time since infection, is likely to have a greater influence on the model predictions than any difference between acute and chronic phase transmission. However, there are two reasons why this is more difficult to allow for. First of all the result shown in Figure 8 assumes a relationship between survival and set-point viral load based on only one small study and better data are needed if this is to be made the basis for modelling the epidemic. Secondly, including this variation would probably need a model that includes the distribution of set-point viral loads explicitly and the structure of the models that are currently used does not allow for this. If this were to be explored further, the first priority would be to consider models in which the variation in the set-point viral load is included explicitly and comparisons made with a model in which this variation is set to zero.

We conclude, therefore, that increased transmission during the acute phase is unlikely to change the model predictions significantly for several reasons:

1. The acute phase duration is likely to be of the order of two to three weeks or about 0.3% of the total disease duration.
2. If transmission during the acute phase is sufficiently high for transmission during this short time to be important, then *R*_0_ must be correspondingly low and the reduction in *R*_0_ needed for elimination is correspondingly less.
3. There is strong evidence that transmission saturates above a viral load of about 4 to 5 logs mitigating the impact of even very high viral loads during the acute phase.
4. A more important limitation of the current model structures, is that variation in the set-point viral load is not included and this should be explored further.
5. The short duration of the acute phase means that it can only ever make a significant contribution to transmission if the rate of partner changes is much higher than is generally observed to be the case.

# 7.0 Appendix 1 Transforming and fitting the density function of viral load

Let *f*(*v*) be the probability density function of *v*, the logarithm of the viral load to the base ten. We are going to apply a Box-Cox transform to *v* so that the transformed variable *w* is

where $\bar{v}$ is the geometric mean of the data. The density function of *w* is then

$$g(w)=f(v)\frac{dv}{dw}$$

where

We first transform the data using Equation and vary the parameter *λ* to ensure that the transformed data is symmetrical with a skewness of zero. We then fit a normal distribution to the transformed data and carry out the reverse transform to get the fit shown in Figure 2.

For the data from Orange Farm, South Africa^11^ the Box-Cox parameter is *λ* = 2.580 and we then fit the transformed data to a normal distribution with a mean of 20.8 and a standard deviation of 9.4.

To determine the density function for the data in Study 1[^22^](#_ENREF_22) we assume that the distribution is the same as for the Orange Farm data. To determine the density function for the data in Study 2 we apply the Box-Cox parameter for Orange Farm to these data and then fit the observed cumulative distribution function to a normal cumulative distribution function. In both cases, once we have the density function we can then work out the mean value of the viral load within each range as given in the papers.

# 7.1 Appendix 2 Random versus regular testing

Let the relative risk of infection vary with time since infection as *RR*(*t*) Then under random testing at a rate *ρ* year, the reduction in the overall transmission will be

while under regular testing at an interval of *τ* years the reduction in overall transmission will be

Since we have estimates of the relative risk of transmission, given that a person is alive, for different stages of infection, we approximate *RR*(*t*) with an appropriate step function.

# 7.2 Appendix 3 *R*0 and the growth rate for an *n*-stage model

We want to introduce an acute phase but also keep four chronic stages in order to ensure that the survival distribution approximates the observed Weibull distribution with a shape parameter of abut 2 reasonably well. The equations for this model are (keeping the total population constant)

, *i* = 2 to *n*

where *I*_0_ refers to the proportion of people that are susceptible people and *Ii* to the proportion in each successive stage of infection. The infectiousness of each stage is determined by *βi* and the mean duration of each stage is 1/*ρi*. From Equations to it follows that

$$R_{0}=\sum_{i=1}^{n} \frac{\beta_{i}}{\rho_{i}}$$

During the initial exponential growth of the epidemic at a rate *r* we have *I*_0_ ≈ 1 and

*i* = 0 to *n*

from which it follows that

$$\sum_{i=1}^{n} \beta_{i}r_{i}=1$$

with

$r_{i}=\prod_{j=0}^{i-1} \frac{\rho_{j}}{r+\rho_{j}}$.

We can now determine the relationship between the relative risk of infection in each stage and *R*_0_ while constraining the overall growth rate, *r*, as follows. We first set the duration of each stage 1/*ρi* and the relative infectiousness of each stage *βi*/*β*0, for *i* = 1 to *n*, and then vary *β*0 to get the required value of the initial growth rate *r*. Using Equation we then calculate *R*_0_ directly.

# 8.0 References

1. Granich RM, Gilks CF, Dye C, De Cock KM, Williams BG. Universal voluntary HIV testing with immediate antiretroviral therapy as a strategy for elimination of HIV transmission: a mathematical model. Lancet. 2009; **373**(9657): 48-57.

2. Lugada E, Levin J, Abang B, Mermin J, Mugalanzi E, Namara G, et al. Comparison of home and clinic-based HIV testing among household members of persons taking antiretroviral therapy in Uganda: results from a randomized trial. J Acquir Immune Defic Syndr. 2010; **55**(2): 245-52.

3. Granich R, Muraguri N, Doyen A, Garg N, Williams BG. Achieving Universal Access for HIV and TB: potential prevention impact of an integrated multi-disease prevention campaign in Kenya. In preparation. 2011.

4. Nakibinge S, Maher D, Katende J, Kamali A, Grosskurth H, Seeley J. Community engagement in health research: two decades of experience from a research project on HIV in rural Uganda. Tropical medicine & international health : TM & IH. 2009; **14**(2): 190-5.

5. Marseille E, Kahn JG, Pitter C, Bunnell R, Epalatai W, Jawe E, et al. The cost effectiveness of home-based provision of antiretroviral therapy in rural Uganda. Appl Health Econ Health Policy. 2009; **7**(4): 229-43.

6. Harling G, Orrell C, Wood R. Healthcare utilization of patients accessing an African national treatment program. BMC Health Serv Res. 2007; **7**: 80.

7. Guthrie T, Ndlovu N, Muhib F, Hecht R, Case K. The long-term costs of HIV-AIDS in South Africa. Washington, D.C.: Results for Development; 2010.

8. Thomas LS, Manning A, Holmes CB, Naidoo S, van der Linde F, Gray GE, et al. Comparative costs of inpatient care for HIV-infected and uninfected children and adults in Soweto, South Africa. J Acquir Immune Defic Syndr Hum Retrovirol. 2007; **45**(4): 410-6.

9. Levy A, Johnston K, Annemans L, Tramarin A, Montaner J. The impact of disease stage on direct medical costs of HIV management: a review of the international literature. Pharmacoeconomics. 2010; **28 Suppl 1**: 35-47.

10. USAID. 2011 [cited; Available from: http://www.aidstar-one.com/

11. WHO. 2011 [cited; Available from: http://www.who.int/hiv/amds/en/

12. Marseille E, Kahn JG, Pitter C, Bunnell R, Epalatai W, Jawe E, et al. The Cost Effectiveness of Home-Based Provision of Antiretroviral Therapy in Rural Uganda. Appl Health Econ Health Policy. 2009; **7**(4): 229-43 10.2165/11318740-000000000-00000.

13. Braitstein P, Ayuo P, Mwangi A, Wools-Kaloustian K, Musick B, Siika A, et al. Sustainability of first-line antiretroviral regimens: findings from a large HIV treatment program in western Kenya. J Acquir Immune Defic Syndr. 2010; **53**(2): 254-9.

14. WHO/UNAIDS/UNICEF. Towards Universal Access: Scaling up priority HIV/AIDS interventions in the health sector. 2010 [cited; Available from: http://www.who.int/hiv/pub/2009progressreport/en/

15. Montaner J. Highly active antiretroviral therapy and HIV transmission - Author's reply. Lancet. 2006; **368**(9548): 1647.

16. Powers KA, Ghani AC, Miller WC, Hoffman IF, Pettifor AE, Kamanga G, et al. The role of acute and early HIV infection in the spread of HIV and implications for transmission prevention strategies in Lilongwe, Malawi: a modelling study. Lancet. 2011.

17. Epstein H, Morris M. Concurrent partnerships and HIV: an inconvenient truth. Journal of the International AIDS Society. 2011; **14**: 13.

18. Peterman TA, Stoneburner RL, Allen JR, Jaffe HW, Curran JW. Risk of human immunodeficiency virus transmission from heterosexual adults with transfusion-associated infections. Journal of the American Medical Association. 1988; **259**(1): 55-8.

19. Quinn TC, Wawer MJ, Sewankambo N, Serwadda D, Li C, Wabwire-Mangen F, et al. Viral load and heterosexual transmission of human immunodeficiency virus type 1. Rakai Project Study Group. N Engl J Med. 2000; **342**(13): 921-9.

20. Tovanabutra S, Robison V, Wongtrakul J, Sennum S, Suriyanon V, Kingkeow D, et al. Male viral load and heterosexual transmission of HIV-1 subtype E in northern Thailand. J Acquir Immune Defic Syndr. 2002; **29**(3): 275-83.

21. Fideli US, Allen SA, Musonda R, Trask S, Hahn BH, Weiss H, et al. Virologic and immunologic determinants of heterosexual transmission of human immunodeficiency virus type 1 in Africa. AIDS Res Hum Retroviruses. 2001; **17**(10): 901-10.

22. Attia S, Egger M, Muller M, Zwahlen M, Low N. Sexual transmission of HIV according to viral load and antiretroviral therapy: systematic review and meta-analysis. AIDS. 2009; **23**(11): 1397-404.

23. Melo MG, Santos BR, De Cassia Lira R, Varella IS, Turella ML, Rocha TM, et al. Sexual transmission of HIV-1 among serodiscordant couples in Porto Alegre, southern Brazil. Sex Transm Dis. 2008; **35**(11): 912-5.

24. Castilla J, Del Romero J, Hernando V, Marincovich B, Garcia S, Rodriguez C. Effectiveness of highly active antiretroviral therapy in reducing heterosexual transmission of HIV. J Acquir Immune Defic Syndr Hum Retrovirol. 2005; **40**(1): 96-101.

25. Ragni MV, Faruki H, Kingsley LA. Heterosexual HIV-1 transmission and viral load in hemophilic patients. J Acquir Immune Defic Syndr Hum Retrovirol. 1998; **17**(1): 42-5.

26. Operskalski EA, Stram DO, Busch MP, Huang W, Harris M, Dietrich SL, et al. Role of viral load in heterosexual transmission of human immunodeficiency virus type 1 by blood transfusion recipients. Transfusion Safety Study Group. Am J Epidemiol. 1997; **146**(8): 655-61.

27. Garcia PM, Kalish LA, Pitt J, Minkoff H, Quinn TC, Burchett SK, et al. Maternal levels of plasma human immunodeficiency virus type 1 RNA and the risk of perinatal transmission. Women and Infants Transmission Study Group. N Engl J Med. 1999; **341**(6): 394-402.

28. Celum CL, Robinson NJ, Cohen MS. Potential effect of HIV type 1 antiretroviral and herpes simplex virus type 2 antiviral therapy on transmission and acquisition of HIV type 1 infection. J Infect Dis. 2005; **191**(Suppl 1): S107-14.

29. Lingappa JR, Hughes JP, Wang RS, Baeten JM, Celum C, Gray GE, et al. Estimating the impact of plasma HIV-1 RNA reductions on heterosexual HIV-1 transmission risk. PLoS ONE. 2010; **5**(9): e12598.

30. Williams BG, Korenromp EL, Gouws E, Schmid GP, Auvert B, Dye C. HIV Infection, antiretroviral therapy, and CD4+ cell count distributions in African populations. J Infect Dis. 2006; **194**(10): 1450-8.

31. Baeten JM, Kahle E, Lingappa JR, Coombs RW, Delany-Moretlwe S, Nakku-Joloba E, et al. Genital HIV-1 RNA Predicts Risk of Heterosexual HIV-1 Transmission. Science translational medicine. 2011; **3**(77): 77ra29.

32. Box GEP, Cox DR. An analysis of transformations. Journal of the Royal Statistical Society Series B-Methodological. 1964; **26**: 211-46.

33. Donnell D, Baeten JM, Kiarie J, Thomas KK, Stevens W, Cohen CR, et al. Heterosexual HIV-1 transmission after initiation of antiretroviral therapy: a prospective cohort analysis. Lancet. 2010; **375**(9731): 2092-8.

34. Fiebig EW, Wright DJ, Rawal BD, Garrett PE, Schumacher RT, Peddada L, et al. Dynamics of HIV viremia and antibody seroconversion in plasma donors: implications for diagnosis and staging of primary HIV infection. AIDS. 2003; **17**(13): 1871-9.

35. Robb M. RV217: The early capture HIV cohort study (ECHO). Propsective identification of HIV infection prior to acute viraemia among high-risk populations. Poster Abstract 404. Presented at the Keystone Symposium. Whistler, British Columbia, Canada; 2011.

36. Pilcher CD, Joaki G, Hoffman IF, Martinson FE, Mapanje C, Stewart PW, et al. Amplified transmission of HIV-1: comparison of HIV-1 concentrations in semen and blood during acute and chronic infection. AIDS. 2007; **21**(13): 1723-30.

37. Cohen MS, Shaw GM, McMichael A, J., Haynes BF. Acute HIV-1 Infection. N Engl J Med. 2011; **364**(20): 1943-54.

38. Wawer MJ, Gray RH, Sewankambo NK, Serwadda D, Li X, Laeyendecker O, et al. Rates of HIV-1 Transmission per Coital Act, by Stage of HIV-1 Infection, in Rakai, Uganda. J Infect Dis. 2005; **191**(9): 1403-9.

39. Hollingsworth TD, Anderson RM, Fraser C. HIV-1 Transmission, by Stage of Infection. J Infect Dis. 2008; **198**(5): 687-93.

40. Abu-Raddad L, Longini IM, Jr. No HIV stage is dominant in driving the HIV epidemic in sub-Saharan Africa. AIDS. 2008; **22**: 1055–61.

41. Auvert B, Ballard R, Campbell C, Carael M, Carton M, Fehler G, et al. HIV infection among youth in a South African mining town is associated with herpes simplex virus-2 seropositivity and sexual behaviour. AIDS. 2001; **15**(7): 885-98.

42. Plummer FA, Ball TB, Kimani J, Fowke KR. Resistance to HIV-1 infection among highly exposed sex workers in Nairobi: what mediates protection and why does it develop? Immunol Lett. 1999; **66**(1-3): 27-34.

43. Arnaout RA, Lloyd AL, O'Brien TR, Goedert JJ, Leonard JM, Nowak MA. A simple relationship between viral load and survival time in HIV-1 infection. Proceedings of the National Academy of Sciences USA. 1999; **96**(20): 11549-53.

44. Williams BG, Gouws E. The epidemiology of human immunodeficiency virus in South Africa. Philos Trans R Soc Lond B Biol Sci. 2001; **356**(1411): 1077-86.
